# MicroRNAs in Prostate Cancer Liquid Biopsies: Early Detection, Prognosis, and Treatment Monitoring

**DOI:** 10.3390/cells15010083

**Published:** 2026-01-04

**Authors:** Seyyed Mohammad Yaghoubi, Erfan Zare, Sina Jafari Dargahlou, Maryam Jafari, Mahdiye Azimi, Maedeh Khoshnazar, Solmaz Shirjang, Behzad Mansoori

**Affiliations:** 1 Department of Plant, Cell and Molecular Biology, Faculty of Natural Sciences, University of Tabriz, Tabriz 5166616471, Iran; m.yaghoubi99@ms.tabrizu.ac.ir; 2 Pharmaceutical Analysis Research Center, Tabriz University of Medical Sciences, Tabriz 5165665811, Iran; 3 Cancer Immunology and Immunotherapy Research Center, Ardabil University of Medical Sciences, Ardabil 5371713111, Iran; 4 Students Research Committee, School of Medicine, Ardabil University of Medical Sciences, Ardabil 5618985991, Iran; 5 Department of Biophysics, Faculty of Advanced Technologies, University of Mohaghegh Ardabili, Namin 5619911367, Iran; 6 Student Research Committee, School of Medicine, Anzali International Campus, Guilan University of Medical Sciences, Rasht 4144666949, Iran; 7 Department of Animal Biology, Faculty of Natural Sciences, University of Tabriz, Tabriz 5166616471, Iran; 8 Department of Developmental Biology, TeMS.C. Islamic Azad University, Tehran 1916893813, Iran; maedehkhoshnazar2005@gmail.com; 9 Genetics, Genomics and Cancer Biology Program, Sidney Kimmel Comprehensive Cancer Center, Jefferson College of Life Sciences, Thomas Jefferson University, Philadelphia, PA 19107, USA; 10 Program in Molecular and Cellular Oncogenesis, The Wistar Institute, Philadelphia, PA 19104, USA

**Keywords:** microRNA, prostate cancer, liquid biopsies, early detection, prognosis, treatment monitoring, biomarkers

## Abstract

Prostate cancer (PCa) is a common malignancy in men worldwide, with incidence projected to rise in the coming years. Traditional screening and diagnostic methods, such as prostate-specific antigen (PSA) testing and biopsy, face limitations in specificity and invasiveness. Circulating microRNAs (miRNAs) have emerged as stable, non-invasive biomarkers obtainable via liquid biopsies (blood, urine, semen) that could transform PCa management. These small regulatory RNAs reflect underlying tumor biology and are detectable at early disease stages, enabling improved early detection when used alongside or in place of PSA. Distinct miRNA expression patterns correlate with tumor aggressiveness. For example, *miR-141* and *miR-375* are elevated in metastatic cases, whereas *let-7* family members and *miR-326* are upregulated in aggressive disease, highlighting their prognostic value. Moreover, dynamic changes in reported miRNAs during therapy provide real-time insights into treatment response. In androgen-deprivation therapy (ADT), oncogenic miRNAs, such as *miR-21* and *miR-125b*, increase upon resistance, whereas a decline in tumor-suppressive miRNAs, such as *miR-23b/-27b*, flags the transition to castration-resistant PCa (CRPC). Similarly, baseline levels of miRNAs (e.g., *miR-200b/c*, *miR-20a*) can predict chemotherapy outcomes. Integrating multi-miRNA panels has demonstrated superior accuracy for risk stratification and monitoring, paving the way for personalized treatment. Although promising, clinical implementation of miRNA-based assays requires further validation, standardization of protocols, and large-scale prospective studies. Harnessing circulating miRNAs could usher in a new era of precision oncology for PCa, improving early diagnosis, prognostication, and real-time therapeutic guidance.

## 1. Introduction

**Figure 1:**
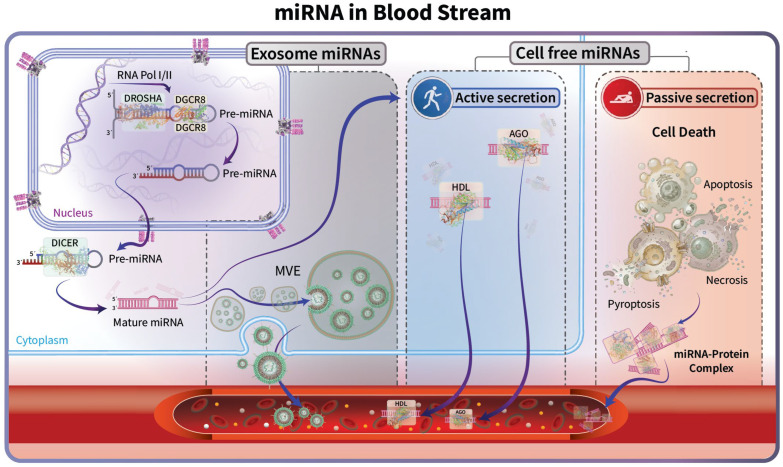
Biogenesis and release of cell-free and exosomal microRNAs in prostate cancer. The (**left panel**) illustrates canonical miRNA biogenesis, beginning with transcription of primary miRNAs (pri-miRNAs) by RNA polymerase I/II, followed by nuclear processing by the DROSHA–DGCR8 complex to generate precursor miRNAs (pre-miRNAs). Pre-miRNAs are exported to the cytoplasm and processed by DICER to form mature miRNAs. A subset of mature miRNAs is selectively sorted into multivesicular endosomes (MVEs) and packaged into intraluminal vesicles, which are released as exosomes into the extracellular space and subsequently enter the systemic circulation. The (**middle panel**) illustrates active secretion, specifically the non-vesicular secretion of cell-free miRNAs, in which mature miRNAs are exported bound to RNA-binding proteins, most notably Argonaute (AGO) complexes, or associated with lipoproteins, such as high-density lipoprotein (HDL). This process protects the miRNAs from extracellular RNase degradation and allows for their direct secretion into the bloodstream. The (**right panel**) illustrates the passive release of miRNAs during cell death processes, including apoptosis, necrosis, and pyroptosis, or therapy-induced cellular stress, resulting in the release of intracellular miRNAs into the blood circulation as protein-bound complexes or within cellular debris. Together, these mechanisms contribute to the pool of circulating miRNAs detectable in the bloodstream and other biofluids in prostate cancer.

Prostate cancer (PCa) is one of the most prevalent malignancies among men globally, and its incidence is projected to rise steadily. Globally, it ranks second in terms of incidence and fifth in cancer-related mortality among men. According to GLOBOCAN estimates, more than 1.2 million new cases were reported in 2018, with higher prevalence observed in developed countries. PCa remains a significant contributor to cancer-associated deaths, and variations in global incidence largely reflect differences in diagnostic testing practices [1]. Approximately 75% of PCa is diagnosed at the localized stage, where the five-year survival rate is close to 100%. Conversely, approximately 10% of men present with distant metastases, where the five-year survival rate drops to 37%. However, even patients with initially localized disease may experience metastatic progression following therapy [2]. The conventional diagnostic approach involves measuring prostate-specific antigen (PSA) in the serum, as the most widely accepted marker for the diagnosis of PCa, in addition to a digital rectal examination (DRE). Despite the usefulness of PSA as a marker, it has limitations, and other serum, urine, and tissue markers are increasingly finding applications [3]. Recent developments in MRI–ultrasound fusion techniques have greatly improved lesion targeting and detection, but prostate biopsy remains the gold standard for diagnosing PCa. Fusion-targeted biopsies increase the detection of clinically significant tumors, enable highly accurate repeat sampling, and are two to three times more sensitive than systematic biopsies. When compared to traditional systematic approaches, these innovations promise to improve diagnostic precision while requiring fewer biopsies [4]. The standard method involves systematic sampling of the prostate using transrectal ultrasound (TRUS)-guided biopsy, which traditionally obtains approximately a dozen tissue cores. However, this method may introduce sampling errors, as well as limitations in the number of core biopsy specimens, which can result in under-detection. Evidence has confirmed the importance of extending the protocol to 20 core biopsies, as there is a significant increase in cancer detection compared to the current protocol, which uses 12 core biopsies. Notably, the expanded protocol addresses the issue of heterogeneity and false negatives more effectively, which are particularly prevalent in systematic biopsies due to their random nature. In settings where multiparametric MRI-guided fusion biopsy was not available, a 20-core systematic biopsy served as an important alternative for improving diagnostic accuracy [5,6]. Collectively, limitations in current diagnostic modalities have intensified the search for more precise, minimally invasive strategies to evaluate PCa. Despite advances in PSA testing, imaging, and biopsy strategies, significant clinical gaps persist in detecting clinically significant PCa and monitoring disease progression over time. PSA lacks sufficient precision to reliably distinguish indolent from aggressive tumors, contributing to overdiagnosis and unnecessary invasive procedures. Although imaging-guided biopsy approaches have improved lesion targeting, they remain invasive, resource-intensive, and poorly suited for repeated longitudinal assessment. These limitations underscore the need for complementary biomarkers that can be measured using minimally invasive and accessible platforms. Liquid biopsy-based biomarkers, particularly circulating microRNAs (miRNAs), offer distinct advantages, including stability in biofluids, ease of serial sampling, and the ability to capture dynamic host-derived molecular changes. Importantly, the stability and detectability of circulating miRNAs in biofluids are directly linked to the biological mechanisms governing their release and extracellular transport. Circulating miRNAs detected by liquid biopsy originate through biologically distinct mechanisms that include both vesicular and non-vesicular pathways. Following canonical biogenesis, mature miRNAs may be selectively incorporated into multivesicular endosomes and secreted as exosomes, where membrane encapsulation confers high stability and facilitates intercellular communication. In parallel, prostate cancer cells actively release miRNAs through non-vesicular pathways, in which mature miRNAs are exported in association with RNA-binding proteins, most notably Argonaute complexes, or bound to lipoproteins such as HDL. These protein- and lipid-associated carriers protect miRNAs from enzymatic degradation, allowing them to persist in the circulation. In addition, passive release of miRNAs occurs during apoptosis, necrosis, pyroptosis, and therapy-induced cellular stress, leading to the extracellular accumulation of intracellular miRNAs as stable protein complexes or within cellular debris. The relative contribution of these pathways shapes the circulating miRNA landscape in prostate cancer and underlies the diagnostic, prognostic, and treatment response utility of both cell-free and exosomal miRNAs in liquid biopsy applications [7,8,9,10] (Figure 1).

With advancements in imaging and biopsy, there has been a growing trend in the literature toward the use of molecular and genomic techniques, based on tissues, blood, and urine, to enhance diagnostic, prognostic, and risk assessment capabilities. Among the newly discovered biomarkers, miRNAs are the most widely explored. These short, highly conserved non-coding RNAs regulate gene expression through post-transcriptional silencing and are remarkably stable in biological fluids, making them well-suited for non-invasive testing. Early studies have demonstrated that circulating miRNAs, such as *miR-141*, exhibit surprisingly high stability in blood and can effectively distinguish between patients with PCa and healthy controls, supporting their utility as potential biomarkers. Numerous miRNAs are consistently dysregulated in PCa relative to nonmalignant tissues and differentiate indolence from aggressive PCa. This includes several miRNAs that have been repeatedly identified across independent analyses, such as *miR-21*, *miR-375*, and *miR-141*, which consistently show an elevation in PCa tissue and serum, with some also linked to disease aggressiveness or progression. Given the growing incidence of PCa, even in developing centers lacking sophisticated imaging or biopsy technology, the availability of molecular technology becomes more urgent. This is particularly relevant in low- and middle-income countries, where limited access to advanced diagnostics, high costs, transportation barriers, and disparities in care impede timely evaluation; therefore, simple, low-cost molecular assays may help bridge critical gaps in resource-restricted environments. Liquid biopsy platforms have expanded beyond circulating tumor cells to include circulating miRNAs and other molecular analytes, offering a feasible and scalable means to capture tumor biology across diverse clinical settings. By enabling molecular profiling through easily obtainable biofluids, such as serum and urine, circulating miRNAs provide new opportunities to improve early detection, refine prognostic assessment, and support dynamic monitoring of disease progression or treatment response. Furthermore, urinary and exosomal miRNAs broaden the diagnostic landscape. This approach provides stable and readily detectable markers that may surpass the performance of current urine-based tests, such as PCA3 [11,12]. The aim of this review is to highlight how emerging research trends demonstrate the growing potential of miRNAs as clinically relevant biomarkers in prostate cancer.

## 2. miRNAs in Early Detection of PCa

### 2.1. miRNAs in Blood Samples

Circulating miRNAs are remarkably stable in biofluids, such as serum and plasma, making them attractive candidates for the development of non-invasive biomarkers in PCa. Several studies have demonstrated the presence of distinct miRNAs in the circulation, whose expression levels are elevated in patients with PCa compared to normal individuals. In this regard, a combination of *miR-152-3p*, *miR-98-5p*, *miR-326*, and *miR-4289* has been demonstrated to be elevated in the plasma of PCa patients, portraying a high level of diagnostic efficiency as this four-miRNA plasma signature achieves a specificity and sensitivity corresponding to an Area Under the Curve (AUC) of 0.88 when distinguishing PCa patients from healthy controls. Notably, this upregulation has been reproduced across two independent patient cohorts. For *miR-98* and *miR-326*, elevated plasma levels have been reported as relatively enriched in PCa cohorts compared with several other malignancies; however, this pattern is not universal and does not establish strict disease specificity. Furthermore, early findings suggest that *miR-98* may also hold prognostic relevance, having been reported as upregulated in recurrent cases following radical prostatectomy, highlighting its potential dual role in diagnosis and disease monitoring [13].

Apart from the diagnostic power, different miRNAs expressed in the circulation have been investigated for biological relevance and mechanism-based roles in PCa. Recent findings highlight that several circulating miRNAs, such as *miR-9-3p*, *miR-330-3p*, and *miR-345-5p*, are significantly overexpressed in the serum of PCa patients compared to healthy individuals, reinforcing their potential oncogenic roles and diagnostic utility. Although these miRNAs were consistently elevated in small patient populations, there is a lack of multicenter validation. Additionally, analytical platform variability currently precludes the definitive designation of these markers with high confidence. Further research is necessary to establish the role played by each miRNA and to clarify how their circulating levels relate to tumor biology, disease status, and therapeutic response, particularly in the context of *miR-345-5p*, which has shown promise as both a diagnostic and treatment response biomarker [14].

Another notable example that targets the PSA 3′ UTR directly is *miR-183*. This miRNA upregulates PSA at the mRNA and protein levels. Clinical evidence also indicates a positive correlation between the levels of prostatic *miR-183* in patients and the levels of circulating PSA, suggesting a regulatory role for *miR-183* in the secretion of PSA. As *miR-183* does not alter androgen receptor (AR) transcript or protein levels, it indicates that this effect is independent of AR signaling. Furthermore, elevated *miR-183* levels correlate with higher WHO grade, increased cell proliferation, and earlier clinical progression, reinforcing its potential oncogenic role and relevance in PSA-based early detection [15]. Such findings provide a mechanistic explanation that may shed light on how specific miRNAs actively regulate known clinical biomarkers, rather than merely serving as passive indicators of disease progression.

Moreover, *miR-19a-3p* suppresses the migration, invasion, as well as the epithelial–mesenchymal transition (EMT) in PCa cells through inhibiting SOX4 expression. The ability of *miR-19a-3p* to regulate the protein levels of EMT markers underscores its significance in the context of PCa research. Although the following evidence primarily represents functionally and mechanistically oriented research, the observations are cited to demonstrate how biologically active miRNAs within the bloodstream can impact tumor biology. While there may be a potential role in the clinic, it appears that *miR-19a-3p* downregulates pro-invasive markers, such as *MMP2*, *MMP9*, *N-cadherin*, *Vimentin*, and *α-SMA*, and upregulates the epithelial marker *E-cadherin*, indicating its impact on tumor aggressiveness through coordinated mechanisms. Furthermore, the translational significance of these miRNAs as a fluid-phase biomarker remains in its early stages because most of the available literature has been conducted in in vitro studies. Given the fact that SOX4 has an established oncogenic role across multiple malignancies and in EMT processes, the ability of *miR-19a-3p* to suppress *SOX4* further underscores its relevance as a promising therapeutic and biomarker candidate in PCa [16].

Among circulating miRNAs, *miR-141* is consistently upregulated in PCa, and its level in urinary pellets shows a significant correlation with Gleason score (GS), indicating its potential as a non-invasive marker for disease aggressiveness. In contrast, its levels in urinary exosomes show no significant differences between patients and healthy controls when isolated by differential centrifugation, although some studies report a trend toward upregulation. Such differences may well represent a methodology disparity in isolating extracellular vesicles (EVs), where differential centrifugation may co-isolate variable populations of vesicles, thus affecting tumor-derived miRNA expression levels. These methodological inconsistencies highlight that evidence regarding its exosomal levels remains mixed, and further standardized studies are needed to clarify its utility as an exosome-based biomarker [17,18]. The presence of strong *miR-141* RNA in urinary pellets but not in exosomes emphasizes a critical technical challenge. The nature of a biomarker assay, which relies on an experimental protocol used for isolating these EVs, such as differential centrifugation, polymer precipitation methods, and size-exclusion chromatography, remains a major source of variability at present. Regarding urinary *miR-141*, these variations in exosomal analysis hinder the reliable translation of this biomarker into a clinically feasible tool. To gauge the challenges associated with the abundance and the lengthy processes of PCR techniques, new photoelectrochemical biosensing technologies have emerged. Based on the principle of redox cycling using singlet oxygen, these biosensors have the capability to specifically identify tumor-associated miRNAs, such as *miR-141*, directly from plasma samples at a detection limit of approximately 0.62 picomolar (pM) within a few minutes [19]. Similarly, *miR-375* is frequently upregulated in PCa tissue and in circulation, with increased expression associated with higher GS, advanced pathological stage, and regional lymph node metastases, highlighting its relevance as both a diagnostic and prognostic biomarker. Interestingly, *miR-375* appears to play a dual role in PCa, acting either as an oncogenic microRNA (oncomiR) or a tumor-suppressive microRNA depending on the cellular context. Measuring circulating miRNAs in blood does not reflect tumor biology alone. Instead, the signal represents a mixture of sources, including tumor cells, surrounding stromal cells, treatment effects, and normal physiological processes; therefore, studies that rely solely on these measurements have an inherent limitation when interpreting tumor-specific miRNA expression. In the androgen-responsive cell line (LNCaP), which reflects a clinically localized, therapy-naïve tumor, high *miR-375* expression correlates with oncogenic activity. In contrast, in androgen-independent cells (DU145 and PC-3), lower expression may correspond to tumor-suppressive effects. This seeming paradox can be explained in part by differences in disease stage, androgen responsiveness, and independence in disease phases, where *miR-375* acts to promote proliferation in androgen-sensitive but potentially has a growth-inhibitory role in androgen-independent disease via different targets. Mechanistically, *miR-375* influences key pathways, including cell cycle regulation, notably targeting *CCND2*, thereby affecting proliferation and progression of PCa. Justification for this bifunctional role is based on its distinct targets and tumor microenvironments. In an androgen-sensitive setting, the overexpression of *miR-375* can promote proliferation through its targets, such as *CCND2*. Alternatively, in an advanced, androgen-independent setting, its tumor suppressor role may involve the suppression of pro-metastatic pathways such as the Yes-associated protein 1 (YAP1) pathway, mirroring a cell’s attempt to regulate excessive plasticity and invasiveness. The microenvironment-dependent role of *miR-375* is also evident in its participation in the ZEB1/miR-375/YAP1 pathway, where it suppresses invasion and metastasis by inhibiting YAP1, as well as other pathways that regulate the epithelial-to-mesenchymal/epithelial (EMT/MET) transition. This dual context-dependent role underscores the complexity of *miR-375* as a biomarker and potential therapeutic target, warranting further investigation in larger cohorts [20].

By contrast, *miR-326* functions as a tumor suppressor and has been observed to be downregulated in cancer tissues in most studies; at the same time, its plasma levels can be upregulated in PCa, highlighting a potential differential pattern between tissue and circulation. Its downregulation in PCa tissues has been frequently associated with disease progression, later staging, increased tumor size, higher pathological grades, and poorer overall survival. In contrast, upregulation in plasma may reflect disease-specific secretion or export mechanisms. Importantly, a mechanistic explanation for this counterintuitive tissue–plasma inverse correlation may arise from the selective incorporation of miRNA into EVs, active secretion in tumor–host communication, or a characteristic response to stress/treatment rather than passive secretion by tumor cells. Additionally, this discrepancy between tissue and plasma miRNA expression emphasizes the essential point for liquid biopsy analysis that miRNA expression within cells, particularly tumor cells, is not necessarily reflected in plasma miRNA levels. On the contrary, plasma miRNAs might predict preferential secretion by cells via EVs, active secretion as a mechanism of tumor–host interactions, or systemic responses to treatment, inflammation, or physiologic stress. Potential regulatory mechanisms include negative control by long non-coding RNAs, such as HOTAIR, which has been linked to the promotion of metastasis and a poor prognosis in several types of cancer. Monitoring *miR-326* levels may provide additional insight into tumor aggressiveness. It could help identify patients at higher risk of adverse prognosis, further supporting its potential utility as a diagnostic and prognostic biomarker in PCa [13].

*miR-146a-5p* is generally regarded as a tumor suppressive miRNA in PCa. It promotes apoptosis and reduces proliferation in androgen-independent cells by targeting the ROCK/Caspase-3 pathway. In tissue studies, *miR-146a-5p* is downregulated in metastatic PCa and in tumors with high GS, with low expression correlating with higher biochemical relapse rates. Moreover, recent evidence indicates that *miR-146a-5p* levels are also reduced in exosomes derived from cancer-associated fibroblasts (CAFs) under androgen-deprived conditions, contributing to increased EMT, migration, and invasion of PCa cells via the EGFR/ERK pathway. These findings underscore its potential value as a biomarker and therapeutic target, although its role in circulating blood samples remains to be clarified. A key pathway illustrating a relevant biological mechanism involves the following sequence: androgen deprivation therapy (ADT) leads to a reduction in exosomal *miR-146a-5p* derived from CAFs. This loss results in diminished inhibition of EGFR/ERK signaling in tumor cells, thereby promoting EMT and metastasis. Collectively, these findings establish an association between reduced *miR-146a-5p* levels and poor disease progression in patients who do not respond to ADT. Restoration of *miR-146a-5p* expression, including through exosomal delivery, may represent a novel strategy to limit metastatic progression in patients receiving androgen-deprivation therapy [21,22]. Based on a meta-analysis of 13 studies including 2080 participants, circulating *miR-146a-5p* alone shows limited diagnostic value. Although the pooled diagnostic odds ratio was 3.54, both sensitivity and specificity remained below 70% (65.5% and 64.9%, respectively), indicating that *miR-146a-5p* is not sufficient as a standalone biomarker to distinguish prostate cancer from benign conditions [23].

Together, these findings highlight that both oncogenic and tumor-suppressive miRNAs shape the complex molecular landscape of prostate cancer and that diagnostic performance improves when molecular profiling is integrated with physiological tumor information using multimodal approaches. Notably, combining miRNA levels in blood-derived extracellular vesicles, including *miR-93-5p* and *miR-151a-5p*, with MRI-based radiomic features such as texture and signal intensity has achieved diagnostic AUC values of up to 0.95 for high-grade prostate cancer, markedly outperforming standard clinical parameters, including PSA and expert radiographic interpretation [24]. Among the miRNAs discussed in this section, *miR-141* and *miR-375* have demonstrated the most substantial evidence for association with aggressive disease, whereas a plausible biological rationale supports *miR-326*, *miR-19a-3p*, and *miR-146a-5p* but has more limited clinical validation. The non-invasive use of circulating miRNAs has potential applications in the management of PCa. However, to improve patient characterization, a combined miRNA signature profiling should be considered, which warrants further studies.

### 2.2. miRNAs in Urine Samples

*miR-145* and *miR-141* (as discussed in detail in Section 2.1) are significantly increased in urinary EVs and exosomes of PCa patients with higher GSs. In particular, the levels of *miR-145* in urinary EVs were strongly increased in patients with GS ≥ 8 compared with those in patients with scores ≤ 7. In addition, the combination of urinary extracellular vesicle *miR-145* and serum PSA improved the separation power between PCa and benign prostatic hyperplasia (BPH) over the use of PSA alone (AUC, 0.863 vs. 0.805). While the level change in *miR-141* tends to be increased in urinary exosomes, the statistical significance depends on the isolation procedure, suggesting the procedure affects the detectability. Such variability highlights an essential intricacy in the methodology of studies analyzing urine miRNA: both pre-analytical sample processing and fraction and vesicle isolation methods can significantly impact miRNA concentrations in urine. The increased levels in urinary EVs may represent the secretion through a vesicle-enabled process in tumor cells, possibly suggesting a role in the elimination of the tumor-suppressive action of miRNAs, or could represent long-range changes in the system characteristic of the mesenchymal–epithelial transition during metastasis. These data strongly support the use of urinary miRNAs, and particularly the role of *miR-145*, in the search for minimally invasive biomarkers [18,25]. Consequently, following the establishment of the diagnostic associations, various studies have been conducted to explore the biological significance of these miRNAs expressed in urine. *miR-145* is a well-established tumor-suppressive miRNA that has been shown in other cancers to inhibit proliferation, migration, invasiveness, and EMT. It is expressed at a lower level in the tumor tissue than in adjacent non-tumor tissue. At the functional and molecular levels, the roles of *miR-145* include the direct targeting of significant oncogenic genes, namely *CTGF* in esophageal squamous cell carcinoma, *FSCN1* in prostate carcinoma, *ANGPT2* and *NEDD9* in renal cell carcinoma, and *Sox2* in choriocarcinomas. The overexpression of *miR-145* reduces the expression of mesenchymal proteins, like N-cadherin, fibronectin, and vimentin. It increases the expression of epithelial proteins, like E-cadherin, thereby reversing the process of EMT and reducing tumor aggressiveness. Although mechanistically sound, it remains unclear whether increased urinary EV concentrations of *miR-145* represent a tumor-specific secretion process or represent a broader stromal-epithelial repatterning in the prostate microenvironment. These findings reveal the complex role of *miR-145* in the anti-tumor process and suggest that its increased level in urinary EVs could represent an anti-tumor effect with potential application in early detection and risk assessment of prostate carcinoma [26]. In urinary EVs, *miR-375* has demonstrated strong individual predictive power for PCa, with a combination of *miR-375* and *miR-574-3p* yielding the best diagnostic performance [27]. *miR-375* plays a significant role in regulating epithelial plasticity by suppressing the invasion and migration of PCa cells through the inhibition of the transcriptional co-activator, YAP1. Importantly, the strong diagnostic accuracy of urinary *miR-375* does not align with its biological role, which suggests that urinary miRNA concentration can serve as a surrogate marker of disease load rather than a purely tumor suppressor function. Significantly, despite its role in suppressing the pathogenic characteristics and primary cells in the resultant tumors, the circulating levels of *miR-375* are positively correlated with the circulating levels of circulating tumor cells (CTCs) in the metastatic setting, indicating that high urinary and plasma concentrations reflect the extent of disseminated CTCs [28] *miR-574-3p* is consistently detected at higher concentrations in the urine of men with PCa compared with controls, reinforcing its utility as part of a multi-miRNA panel for non-invasive early detection [27].

**Figure 2:**
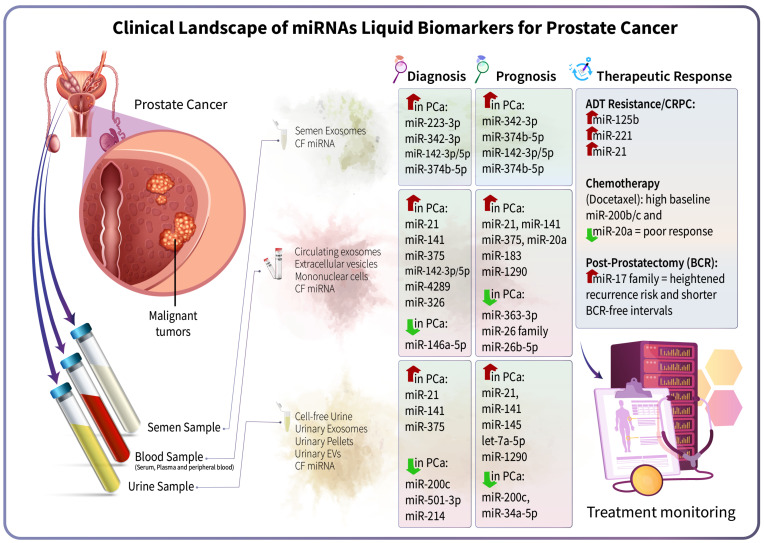
Circulating miRNA biomarkers in PCa liquid biopsies. Liquid biopsy approaches in prostate cancer enable the detection of microRNAs (miRNAs) from blood, urine, and semen, providing clinically relevant information for disease diagnosis, prognosis, and therapeutic response. In semen, miRNAs are detected as cell-free miRNAs and within semen-derived exosomes and extracellular vesicles. In blood samples, including plasma, serum, and peripheral blood, miRNAs are identified as cell-free circulating miRNAs, within circulating exosomes and extracellular vesicles, and within mononuclear cells. In urine, miRNAs are present as cell-free miRNAs, urinary exosomes, urinary pellets, and urinary extracellular vesicles. These liquid biopsy-derived miRNA profiles have applications in prostate cancer detection, prognostic assessment, and treatment monitoring, including androgen deprivation therapy (ADT) resistance and castration-resistant prostate cancer (CRPC), chemotherapy response, and post-prostatectomy biochemical recurrence (BCR). Red arrows indicate increased miRNA expression, whereas green arrows indicate decreased miRNA expression.

Several other urinary miRNAs are altered in PCa. *miR-21* is significantly upregulated in both urinary pellets and exosomes, with higher levels observed in both non-metastatic and metastatic PCa compared with BPH [18]. Functionally, *miR-21* promotes tumorigenesis by suppressing multiple tumor suppressor genes that usually restrain proliferation, invasion, and survival, including *PTEN*, *PDCD4*, and *SPRY1*. Downregulation of *PTEN* reduces apoptosis and enhances angiogenesis through the HIF-1α signaling pathway, while suppression of *PDCD4* and *SPRY1* further facilitates tumor cell proliferation, migration, and invasion. *miR-21* also interacts with AR signaling and contributes to EMT, enhancing PCa progression [29]. The ultimate consequence of such targeting is the induction of an aggressive tumor cell phenotype with resistance to therapy and metastatic capability, thus justifying the correlation with poor prognosis. Regarding urinary miRNAs, *miR-21* exhibits a relatively consistent level of detectability across different urine fractions, making it a more reliable marker than other candidate markers with less stable concentrations in bodily fluids. *miR-483-5p* is found at a higher level in the cell-free urine fraction of patients with PCa than in controls, and can serve as a potential biomarker for PCa detection. Additionally, it can be isolated from voluntarily voided urine samples without the need for exosome purification, making it easier to use in prospective medical studies. From a functional perspective, *miR-483-5p* has been reported to play a crucial role in the processes of angiogenesis, cell proliferation, invasion, and metastasis in various types of carcinomas. The availability of this level of *miR-483-5p* detection without isolating EVs suggests a potential translatable benefit, but its specificity in relation to established methods using urine remains to be proven. By combining the quantification of urinary levels of *miR-483-5p* and other markers, such as the PSA, the sensitivity and specificity of the diagnosis could be improved [30]. *miR-1290* is significantly increased in urinary EVs of PCa patients compared with BPH patients, with even higher levels observed in metastatic PCa. Although its predictive value for PCa in Receiver Operating Characteristic (ROC) analysis is not reported as significant, the upregulation of *miR-1290* in UEVs reflects its potential involvement in tumor progression and systemic circulation changes. Such an observation underlines a common shortcoming in existing studies on urinary miRNAs, wherein concurrent diagnostic performance capabilities do not necessarily accompany a robust biological correlation. The detection of *miR-1290* in UEVs highlights the value of urine-derived EVs as a stable source of miRNAs, protected from degradation. It underscores their utility for the discovery of minimally invasive biomarkers [25]. *miR-126-3p* is also overexpressed in urinary EVs of PCa patients compared to biopsy-negative men. Logistic regression analysis adjusted for age and PSA identified *miR-126-3p* as a significant predictor of PCa, with better sensitivity and specificity than serum PSA. Notably, its expression was not associated with GS, suggesting its potential as an early detection biomarker independent of tumor grade. Such properties make *miR-126-3p* one of the more promising urinary miRNAs in clinical prospects despite the need for validation in larger study populations. The stability of miRNAs within UEVs, combined with the non-invasive collection of urine, particularly after digital rectal examination, further highlights *miR-126-3p* as a promising candidate for clinical biomarker development [31] (Figure 2).

*miR-34a-5p*, *miR-143-3p*, *miR-501-3p*, *miR-214*, and *miR-92a-1-5p* were found to be significantly decreased in urinary exosomes of PCa patients versus healthy donors, as identified by deep sequencing [32]. *miR-200c* is reduced in urine from both non-metastatic and metastatic PCa groups compared to BPH [33]. *miR-200c* plays a critical role in suppressing tumor invasion, metastasis, and EMT, thereby inhibiting PCa proliferation [34]. Likewise, *miR-196a-5p* is significantly downregulated in urinary exosomes of PCa patients compared to healthy donors, a finding confirmed by RT-qPCR in an independent cohort. *miR-196a-5p* demonstrated the highest sensitivity in distinguishing patients with PCa from healthy controls. Recently, the specific serum miRNA profile formed by *miR-1-3p*, *miR-96-5p*, *miR-148a-3p*, and *miR-375-3p*, wherein the last three are significantly decreased in prostate cancer (PCa) as compared to BPH, has been discovered through high-throughput sequencing [35]. When the geometric mean of the expression levels of *miR-1-3p* and *miR-148a-3p* is examined in conjunction with PSA levels, the AUC value climbs to 0.97, and the values for sensitivity and specificity reach 95.8% and 91.3%, respectively, for the distinction [35]. Among the downregulated urinary miRNAs, *miR-196a-5p* currently exhibits the best diagnostic capability; however, its utility may be limited by a dependency on exosome separation. While the levels in exosomes do not necessarily reflect the expression in tumor tissue due to the preferential secretion of specific miRNAs, the uniform suppression of these levels in urinary exosomes has important diagnostic implications for the early detection of PCa. Moreover, the regulated target genes of the candidate miRNA, such as the ERG and HOX genes, which are involved in the pathophysiology of PCa, provide evidence of its putative role in the disease’s pathophysiology. The candidate miRNA, *miR-501-3p*, was also reduced in the urinary exosomes, and its role in the disease needs further investigation [32] (Figure 2).

As such, urinary miRNAs have unique translational strengths based on both non-invasive access and their relative origin proximity to the prostate, but both analytical heterogeneity and existing competition in urine tests such as PCA3, SelectMDx, and ExoDx impede the translational application of urinary miRNAs [36]. Although a variety of urinary miRNAs possess equal and/or additive diagnostic capabilities in addition to PSA, direct comparative studies are necessary before their application in a translation setting [37].

### 2.3. miRNAs in Semen and Other Biofluids

In semen exosomes, the levels of *miR-142-3p* and *miR-142-5p* differ statistically between PCa patients and healthy controls [38]. These miRNAs can drive tumor progression by activating the canonical Wnt/β-catenin signaling pathway through APC suppression, as demonstrated in other tumor models [39]. When *miR-142-3p* is combined with serum PSA in a diagnostic model, the accuracy of distinguishing PCa from BPH improves, enhancing the efficiency of PSA-based screening and potentially reducing unnecessary biopsies. *miR-223-3p* is overexpressed in semen exosomes of PCa and BPH patients and shows good accuracy in discriminating PCa from combined controls (healthy control + BPH) [38]. When combined with PSA and *miR-142-3p/5p*, it further enhances the accuracy of PCa diagnosis compared to BPH. Reported targets include *TP53*, *HSP90B1*, *CDK2*, *FOXO1*, *CHUK*, *MDM2*, and *IGF1R* [38] (Figure 2).

Another semen exosomal miRNA, *miR-342-3p*, can distinguish PCa with GS 6/7. When combined with PSA, *miR-342-3p* improved the prediction of higher GS, effectively discriminating between men with GS ≥ 7 and those with noncancerous/GS6 cases. *miR-342-3p*’s targets include *IGF1R*, *E2F1*, *ATF4*, and *IKBKG*, linking it to growth factor and stress response pathways [38]. Similarly, *miR-374b-5p* in semen exosomes can distinguish men with GS ≥ 7 from those with non-cancer/GS6 cases. In combination with *miR-342-3p* and PSA, *miR-342-3p* enhanced predictive accuracy for identifying higher-grade diseases. MiRNAs in exosomes are resistant to enzymatic breakdown, making them optimal non-invasive biomarkers indicative of the health status of the prostate gland. The increased levels of exosome-derived miRNAs *miR-142-3p/5p*, *miR-223-3p*, *miR-342-3p*, and *miR-374b-5p* in semen exosomes correlate with their expression in the prostate tissue and illustrate their functional role in tumorigenic signaling and oncogenic aggressiveness [38]. The functional role of *miR-142-3p/5p* in regulating cellular proliferation and tumor development can be mediated by the canonical Wnt/β-catenin signaling pathway through APC repression [39] (Figure 2).

Table 1 summarizes key miRNAs investigated for PCa detection, outlining their biofluid origins, molecular functions, diagnostic patterns, and diagnostic performance across various studies.

## 3. miRNAs as Prognostic Biomarkers in PCa

PCa morbidity increases significantly once it spreads beyond the prostate (metastasis). The disease most frequently metastasizes to bones, especially the spine, pelvis, and ribs, along with lymph nodes, lungs, and liver. Bone metastases are a hallmark of advanced PCa and are associated with osteoblastic, osteolytic, or mixed phenotypes, reflecting distinct molecular and cellular processes that influence disease progression. Bone involvement often causes pain, fractures, spinal cord compression, and other serious complications. Bone health events have a significant impact on both prognosis and quality of life, with the added risks related to a compromised bony environment, including osteoporosis, potentially exacerbated by ADT. Understanding the metastatic cascade, with a focus on the homing of PCa cells to the bony microenvironment and the stepwise tumor–host dialog, may elucidate a strategy for prevention and treatment targets. Bone status and risks related to osteoporosis must be evaluated in every newly treated PCa patient, and treatment directed against the bony environment may be considered before apparent clinical bony metastases, with a coordinated care team that includes a bone health consultant [43]. Although these principles of patient treatment remain well established, reliable molecular markers do not currently exist that can predict patients at high risk of metastasis before they manifest via radiographic findings. The onset of metastasis marks a shift from localized or locally advanced disease to a more aggressive systemic condition, associated with poorer outcomes and the need for systemic treatments such as ADT, chemotherapy, or targeted therapies, rather than curative local interventions. One pragmatic yet highly predictive method for prognostication involves measuring the total amount of cell-free small RNA (cf-sRNA) and cf-miRNA in both plasma and urine using capillary electrophoresis. PCa patients who progress to mCRPC have significantly higher levels of total cf-miRNA at baseline (836 ng/mL) than those who do not progress (28 ng/mL); measuring total RNA levels is a credible prognostic indicator for assessing the extent of tumor and the efficacy of treatment [44]. This transition is of clinical relevance because men with intermediate-risk or high-risk disease require imaging studies for an evaluation of nodal and/or metastatic disease, and treatment choices may be impacted by disease burden, history of previous treatments, and performance status. For example, the integration of drugs such as abiraterone, enzalutamide, and apalutamide with ADT has demonstrated a favorable effect on overall survival in men with metastatic hormone-naïve PCa, highlighting the need for early identification of men with metastatic disease who may benefit from systemic therapies, thanks to timely diagnostic modalities. Moreover, currently, the care of men with advanced disease relies increasingly on the principles of precision medicine, including the analysis of potential drivers, such as *DDR* or *BRCA2* gene mutations, and molecular markers, such as AR-V7 expression [45].

In clinical practice, PCa is often first suspected due to elevated PSA or abnormal DRE findings. However, definitive diagnosis relies on histological confirmation via biopsy. As noted, serum PSA has long been a non-invasive screening tool, but its limited specificity leads to false positives and overdiagnosis. An abnormal DRE can indicate the need for biopsy, but remains a stronger predictor of cancer than either DRE or transrectal ultrasound. The limitations of PSA screening complicate clinical management, given the heterogeneity of PCa and the need for long-term monitoring. Risk-adapted strategies, including consideration of age, family history, and genetic predisposition, are increasingly recommended to guide biopsy decisions and reduce unnecessary procedures. Repeated biopsies burden patients and may still miss significant tumor areas due to sampling limitations, especially in multifocal cancers such as PCa, ultimately affecting treatment decisions. Emerging imaging techniques, such as multiparametric MRI, are shaping future diagnostic pathways, aiming to improve patient outcomes [46]. In this context, the need for prognostic markers with the capacity to enhance risk assessment in a manner additive to prostate-specific antigen level and imaging persists. In recent years, miRNAs have emerged as promising non-invasive biomarkers for the prognosis of PCa. Over the past decade, extensive research has revealed crucial roles for miRNAs throughout PCa development. These small regulatory molecules influence key cellular processes, including proliferation, apoptosis, cell cycle, EMT, angiogenesis, and metastasis, at every stage from tumor initiation to distant spread. The apparent heterogeneity of PCa, as manifested by the grade of the tumors and their differing biological potential, stresses the need for molecular-based biomarkers, such as miRNAs, to improve the prognostic predictions of disease progression. Furthermore, the long natural history of PCa and the rapidly expanding diagnostic and treatment options, including the use of PSA testing, active surveillance, and newer imaging modalities such as multiparametric MRI and PSMA PET, underscore the importance of available prognostic tests in helping guide the management strategy for individual patients. Despite ongoing efforts, not all miRNAs in circulation have consistently demonstrated prognostic value, underscoring the importance of rigorous validation. Within this paradigm, miRNAs represent a new tool, in addition to other established clinical and pathological factors, for better selection of those patients who may be placed at the highest risk of aggressive disease and avoid treatment for those with indolent disease [47].

### 3.1. MiRNAs and Tumor Aggressiveness

Numerous miRNAs are associated with PCa aggressiveness as indicated by GS and clinical risk scores. For instance, *miR-375* and *miR-141* are prominent markers for high-risk tumors, with higher expression correlating directly with a higher GS. Validation studies conducted on independent sets of cases have demonstrated that the level of *miR-375* is strongly associated with systemic disease characteristics, such as the presence of lymph node metastases and distant metastases. In contrast, the level of *miR-141* is significantly elevated in high-grade disease compared to both intermediate and low-grade disease. Both miRNAs were significantly elevated in tumor tissue versus benign epithelium, indicating a potential correlation with shed miRNAs in the circulation. Among these prognostic miRNAs, *miR-375* and *miR-141* are supported by robust multicenter clinical evidence and are therefore considered higher-confidence prognostic markers (Level 1/Level 2). While the precise mechanisms remain an area of active investigation, it is currently postulated that miRNAs present in the circulation may be secreted by tumor cells through microvesicles, which can communicate with other cells within the tumor microenvironment, potentially affecting disease progression. Taken together, these data indicate the value of circulating levels of *miR-375* and *miR-141* as a non-invasive prognostic tool in combination with established clinical/pathological factors for managing PCa [48]. Conversely, increased levels of *miR-195* and *let-7i* are inversely correlated with GS, tending to be lower in high-grade cancers, consistent with evidence that both miRNAs show significant negative correlations with GS and other adverse pathological features such as surgical margin positivity. Their circulating levels also decrease after prostatectomy, supporting a tumor-associated origin and reinforcing their potential value as noninvasive indicators of less aggressive disease biology [49]. *miR-20a* levels are significantly higher in patients with a GS of 7–10 compared to those with a GS of ≤6, consistent with its association with more advanced tumor stage and high CAPRA scores. Similarly, plasma *miR-21* is significantly increased in patients with intermediate- or high-risk D’Amico scores compared to those with low-risk scores, and *miR-21* is also upregulated in patients with high-risk CAPRA scores. *miR-145* is likewise elevated in intermediate- and high-risk D’Amico groups, consistent with its reported variability and heterogeneous expression patterns in aggressive PCa [40]. A combination panel of four miRNAs (*miR-20a*, *miR-21*, *miR-145*, and *miR-221*) could effectively distinguish high-risk from low-risk PCa patients based on the D’Amico score, while the combination of *miR-21* and *miR-221* differentiated intermediate- from low-risk CAPRA scores [40]. Focusing on pathological GSs, *miR-363-3p* was found to decrease significantly with increasing pathological GS (pGS6, pGS7, pGS8) [50]. Meanwhile, *miR-26b-5p*, *miR-106a-5p*, and *let-7i* could distinguish pGS8 from both pGS6/7, and *miR-18b-5p* and *miR-25-3p* decreased in pGS7 compared to pGS6 [50]. Members of the *miR-26* family (*miR-26a*, *miR-26b*) showed higher levels in lower-risk disease. Increased *miR-26a* expression is inversely correlated with the positivity of surgical margins. This family exhibits a decrease in expression with increasing PCa malignancy. These miRNAs can distinguish between low-risk and intermediate/high-risk patients. These findings align with the broader trend in which *let-7* family members and multiple tumor-suppressive miRNAs (including *miR-26a/b*, *miR-18b-5p*, and *miR-25-3p*) exhibit progressive downregulation with increasing disease severity [50] (Figure 2).

Along with such correlative clinical findings, mechanism-based research has endeavored to clarify how such miRNAs are actively involved in the development of aggressive tumor phenotypes. Mechanistically, oncomiRs and tumor-suppressive miRNAs in PCa target pathways that drive aggressiveness. *miR-221* is an oncomiR that negatively regulates the cell cycle inhibitor p27^Kip1^ and upregulates DVL2 (involved in Wnt signaling for migration). In parallel, *miR-20a* may also promote aggressiveness by inhibiting apoptosis through the targeting of E2F1. At the same time, *miR-21* enhances angiogenesis by suppressing PTEN and activating the AKT/ERK–HIF-1α–VEGF signaling pathway, further supporting its role as a key driver of aggressive PCa biology [40]. *miR-221* may also directly inhibit ARHI (a tumor suppressor gene) by binding to its 3′UTR, and thereby contribute to ARHI downregulation in PCa, a mechanism shown to stimulate cell proliferation and invasion as ARHI loss removes its growth-inhibitory and pro-apoptotic functions [51]. *miR-145* and *miR-331-3p* are implicated in the development and progression of aggressive PC by regulating key cell cycle-related hub genes. Consistent with systems-level analyses identifying both miRNAs as regulators of cell cycle networks in aggressive disease, it is revealed that *miR-331-3p* targets CDCA5 and *miR-145* targets *KIF23* and *CCNA2*; and that ectopic expression of these miRNAs reduces expression of their corresponding hub genes, leading to cell growth inhibition and apoptosis [52] (Figure 2).

Functionally, the *miR-26* family exhibits tumor-suppressive properties. They also regulate EZH2, a key epigenetic regulator, by suppressing its mRNA and protein expression, thereby reducing cellular proliferation in PC. Consistent with this role, both *miR-26a* and *miR-26b* are significantly downregulated in malignant prostate tissues and show further decreases with increasing GS, aligning their loss with tumor aggressiveness. Moreover, experimental overexpression of *miR-26a* and *miR-26b* in PCa cell lines represses EZH2 and leads to measurable reductions in proliferation, supporting their functional relevance in disease progression [50].

The *miR-200* family plays a critical role in CRPC primarily as circulating prognostic biomarkers, where high pre-docetaxel levels of these miRNAs are consistently associated with shorter overall survival (OS) and help stratify these patients as non-responders to docetaxel chemotherapy. This aligns with evidence showing that *miR-200* overexpression is clinically associated with increased metastatic risk and promotes metastatic colonization in vivo. While these miRNAs are generally known to lower EMT, high circulating levels, suggesting poor outcomes, contradict findings from cell line model systems, where downregulation induces drug resistance. Functionally, the association with poor prognosis may be due to the *miR-200* family promoting enhanced metastatic colonization through inhibiting the gene *SEC23A*, which is involved in protein trafficking and regulates the secretion of metastasis-suppressive proteins. These data support a biphasic role for the *miR-200* family, where high expression favors later metastatic colonization despite its EMT-suppressive activity [53,54].

*miR-375* is consistently found upregulated in PCa patients, establishing its significant role as a potential biomarker for detection and prognosis. Its upregulation has been observed in PCa tissue, blood, and particularly in urine, both in urinary pellets and urinary exosomes, suggesting its value in PCa diagnosis, especially when combined with *miR-21*, as this panel yielded the highest AUC = 0.872 for distinguishing PCa patients from healthy subjects. Urinary studies confirm that *miR-375* is significantly increased in both pellets and exosomes of patients with PCa, with higher levels observed in those with intermediate- to high-risk disease, supporting its association with tumor aggressiveness. Functionally, the abnormal expression of *miR-375* has been implicated in the enhanced migration and invasion of tumors; one mechanism is through the repression of *CBX7*, which leads to the activation of pro-metastatic transcriptional programs [18]. Furthermore, *miR-375* also plays a role in MET through a ZEB1-miR-375-YAP1 network, favoring invasion and metastatic disease. Notably, elevated circulating and exosomal *miR-375* levels have been associated with poor overall survival in metastatic CRPC, underscoring its potential as a prognostic biomarker [53].

The expression of *miR-363-3p*, *miR-106a-5p*, and *let-7i* is significantly decreased only in high-risk patients compared to low-risk patients, suggesting that they may serve as potential markers of high-risk disease. Furthermore, *miR-26b-5p*, *miR-106a-5p*, and *let-7i* were able to distinguish both pGS6 and pGS7 from pGS8. Additionally, *miR-605*, *miR-135a*, and *miR-433* form a molecular signature for very high-risk (VHR) aggressive PCa, capable of accurately differentiating VHR cases from low-risk cases and from healthy individuals. Notably, elevated serum levels of *miR-200c* were consistently observed in all VHR PCa patients and are included in the validated circulating miRNA biomarker panel for distinguishing between indolent and aggressive disease [55] (Figure 2).

### 3.2. MiRNAs and Metastasis/Poor Outcome

Several circulating miRNAs have been linked to metastatic disease and poor clinical outcomes. Elevated levels of *miR-21* are observed in CRPC and have also been detected in patients diagnosed with metastatic PCa. In addition, higher pre-docetaxel circulating *miR-21* levels have been associated with shorter overall survival in CRPC patients, although not consistently predictive of chemotherapy response [54]. These patterns indicate that *miR-21* upregulation reflects both disease aggressiveness and poor prognosis [40,54]. *miR-21*′s upregulation contributes to treatment resistance via its inhibition of tumor suppressors such as PTEN and its downstream activation of AKT/ERK signaling and HIF-1α–mediated angiogenesis, mechanisms implicated in promoting invasive and aggressive tumor behavior [40,54,56].

There is a stepwise reduction in the expression levels of *miR-363-3p*, *miR-26b-5p*, and *let-7i* with an increase in pathological grade, with a marked reduction observed in the high-risk group. This implies these miRNAs may serve as potential prognostic markers of aggressive tumor features. Moreover, *miR-26b-5p* and *let-7i* have the potential to distinguish pGS6 and pGS7 from those with pGS8, thereby showing correlation with disease progression. Furthermore, a validated blood miRNA expression panel including *miR-200c*, *miR-605*, miR-135a*, *miR-433*, and *miR-106a* can successfully distinguish very high-risk PCa (VHR PCa) from both low-risk and healthy individuals [50]. These five miRNAs target genes involved in key oncogenic pathways, including tumor suppressors, cell-cycle regulators, and signaling molecules such as PTEN, TP53, RB1, and components of the PI3K, MAPK, and Wnt pathways [40,50,54,55] (Figure 2).

Beyond these, other miRNAs demonstrate diagnostic and progression-related relevance. *miR-139-5p* levels in peripheral blood are significantly higher in patients with PCa than in those with BPH and healthy controls (*p* < 0.001). Higher *miR-139-5p* expression associates with adverse clinicopathologic features, including PSA > 20 ng/mL, pathological stage T3/T4, and GS > 7. *miR-139-5p* can distinguish between BPH and PCa with high accuracy (AUC, 0.936; 95% CI, 0.878–0.993; *p*< 0.001), as well as between PCa and healthy controls (AUC, 0.915; 95% CI, 0.846–0.984; *p* < 0.001). Together, these findings suggest that *miR-139-5p* is a promising non-invasive biomarker for PCa detection and may reflect disease aggressiveness, pending validation in larger cohorts [57]. Furthermore, *miR-139-5p* has already been demonstrated to function with oncogenic and tumor suppressor effects in a cancer type–specific manner, highlighting its potential implication as a modulator of pathways related to tumor progression in a cell lineage-specific manner in cancer types other than PCa. Its potential applications for diagnosis, prognostic purposes, and stratification of risks in PCa, given the associations with high levels of PSA expression, pathological stage, and GS, therefore warrant a detailed exploration of its functional implications in PCa progression and metastasis [57]. Similarly, *miR-21* expression is significantly increased in peripheral blood mononuclear cells (PBMCs) obtained from patients with PCa, which is significantly higher than that observed in healthy controls (*p* < 0.001). High expression of *miR-21* is strongly associated with poor differentiation, advanced stage, and lymph node metastasis (*p* < 0.001) and is more prevalent among individuals with recurrence and/or metastasis. The ROC curve revealed a cut-off value of 0.9, showing 87.5% sensitivity and 85.7% specificity. These results suggest that *miR-21* expression in PBMCs is a promising non-invasive marker for diagnosing PCa and evaluating the probability of recurrence and metastasis. Mechanistically, *miR-21* is postulated to promote tumor progression by inhibiting tumor suppressor genes, such as TPM1 and PDCD4, which play a crucial role in controlling invasion and inducing cell death through apoptosis. Furthermore, the potential role of *miR-21* in influencing cell motility via an actin cytoskeletal rerouting process, as proposed by the gene *MARCKS*, has also been suggested. Moreover, the prognostic significance of increased *miR-21* expression levels in peripheral blood mononuclear cells has been demonstrated, establishing the predictive value of *miR-21* expression levels for the potential recurrence and metastasis of cancer after prostatectomy. This finding defines a potential target role for *miR-21* therapies, such as antagomir-21 [58].

Furthermore, serum *miR-940* levels are higher in cancer patients, particularly in those with clinically significant tumors (GS ≥ 7), compared to healthy individuals. Although *miR-940* expression is reduced in PCa tissues, increased expression in the bloodstream may result from active secretion, which complements tissue expression patterns and is observed with other miRNAs in cancer. This secreted *miR-940* may function to modulate intracellular oncogene activity, such as MIEN1, highlighting a potential regulatory mechanism in tumor progression. The AUC value has been reported to improve when *miR-940* is combined with PSA, rising from 0.75 to 0.818, which demonstrates enhanced diagnostic performance when integrated with conventional biomarkers. These findings further support the utility of circulating miRNAs as non-invasive liquid biopsy markers for disease stratification and prognostic assessment [59] (Figure 2).

A circulating four-miRNA signature (*miR-17*, *miR-20a*, *miR-20b*, *miR-106a*) is significantly increased in high-risk men post prostatectomy (high GS, high pathological Tumor (pT) stage, margin positive, and/or diagnostic PSA > 20 ng/mL) compared to low-risk men. A high expression level is associated with a higher pT stage (pT3+). Still, according to TCGA, it is linked to a shorter time to biochemical recurrence, characterized by increased expression levels of *miR-17*, *miR-20a*, and *miR-106a*. The authors indicate short clinical follow-up within their study, and that while the NanoString technology employed in this study allowed for detection and quantification of members of this miRNA family (with *miR-17* vs. *miR-106a* and *miR-20a* vs. *miR-20b* being two pairs where this could not be accomplished), it is challenging to assign unique effects to each miRNA within this family [60]. *miR-128* levels are significantly decreased in PCa tissue and serum; low *miR-128* is associated with aggressive features (advanced stage, lymph node metastasis, high pre-op PSA, angiolymphatic invasion) and independently predicts shorter biochemical recurrence-free survival. Notably, a strong correlation is observed between the levels of *miR-128* in tissue and serum specimens (r_s = 0.808, *p* < 0.001), suggesting that serum *miR-128* can closely reflect tumor expression levels. Biofunctionally, *miR-128* may suppress tumors in PCa by targeting candidate target genes, including *BMI1*, *E2F3*, *DCX*, and *NTRK3*, which can be involved in cell proliferation, invasion, and apoptosis. Based on these characteristics, it can be concluded that the value of serum *miR-128* may be used as a potential non-invasive indicator for prognostic testing and risk stratification of PCa patients [61]. *miR-34a*, a well-known tumor suppressor, is significantly downregulated in PCa tissue, and its expression is observed to be lower in metastatic tumors than in primary malignancies. Values within urinary samples demonstrated a trend toward reduced *miR-34a* expression in patients with PCa compared to those with BPH, although this result remains to be confirmed on a larger scale. Employing MIRUMIR, reduced *miR-34a* expression approached but did not reach significance concerning survival within PCa (*p* = 0.075) [62]. In contrast, patients with high *miR-21* expression exhibit significantly shorter survival times compared to those with low *miR-21* expression, indicating a poor prognosis in PCa. Elevated *miR-21* levels in PBMCs correlate with aggressive clinicopathological features, including poor differentiation, advanced stage, and lymph node metastasis, high GS, as well as increased risk of recurrence and metastasis, highlighting its potential as a non-invasive prognostic biomarker and therapeutic target [63] (Figure 1).

A previous study has shown that bone metastasis is an independent prognostic factor in patients with PCa. In particular, a low *miR-128* level in both tissue and serum was associated with aggressive features, including lymph node and bone metastases, and independently predicted shorter biochemical recurrence-free survival, highlighting its potential utility as a non-invasive biomarker for assessing metastatic risk and overall prognosis [63]. Metastatic tropism, especially to bone, has distinct miRNA signatures. Reduced serum levels of *miR-218-5p* have been proposed as a biomarker for PCa bone metastasis: low *miR-218-5p* serum levels predict shorter bone metastasis-free survival and correlate with higher PSA and GS compared to non-metastatic or control individuals [64]. Similarly, higher *miR-214* serum levels are positively associated with poorly differentiated tumors (GS > 7) and are significantly upregulated in patients with bone metastasis. This suggests that *miR-214* may serve as a non-invasive biomarker for aggressive PCa and may contribute to tumor progression through targeting *PTEN*, promoting increased invasiveness of PCa cells [41]. Moreover, *miR-181a-5p* is upregulated in the serum exosomes of patients with bone-metastatic PCa; elevated *miR-181a-5p* is significantly associated with bone-metastatic PCa and has been proposed as a diagnostic biomarker for bone metastasis (AUC = 73.8% for bone metastasis). This EV-delivered miRNA may not only reflect tumor aggressiveness but also participate in metastasis by promoting cancer cell migration and invasion, potentially enhanced under hypoxic conditions, highlighting its role in the formation of a pro-metastatic environment [65].

For both CRPC and PCa, there are miRNAs with diagnostic/prognostic value for aggressive PCa. Plasma *miR-107* is upregulated in obese PCa patients and can distinguish PCa samples from controls more accurately than PSA, although it does not surpass its specificity. The expression level is also associated with clinical variables and aggressive factors, proposing a role for this miRNA within personalized medicine [66,67]. *miR-107* is significantly elevated in liquid biopsies from CRPC patients compared to those from non-CRPC patients, and its levels are higher in advanced clinical stages, supporting its potential as a diagnostic and prognostic marker. Separately, plasma exosomal *miR-1290* and *miR-375* were shown to be correlated with poor OS in CRPC patients. The incorporation of *miR-1290* and *miR-375* into OS prediction models increased time-dependent AUC values from 0.66 to 0.73 (*p* = 6.57 × 10^−6^) [42]. *miR-1290* is also an oncogenic miRNA present within plasma exosomes, which is enriched within CRPC and significantly associated with poor OS. *miR-375* and *miR-141* display increased expression within metastatic PCa and associate with high GS and/or lymph node positive status, making them both strong prognosticators [42]. High-throughput NanoString arrays have found that miR-1272, *miR-1247-5p*, *miR-302d-3p*, and *miR-1246* are greatly elevated in PCa, and that miR-1272, *miR-1247-5p*, and *miR-1246* are greatly increased in metastatic PCa compared to localized disease [68]. In addition, EV-based RNA profiling using the TCLN biochip showed serum EV miRNAs (*miR-141*, *miR-375*, and *let-7c*) and urine EV RNAs (*miR-141* and *miR-375*) effectively distinguished PCa patients from controls, with combined serum and urine EV RNA signatures achieving diagnostic AUC values of 0.824 and 0.741, respectively [69]. Another study reports that *miR-1246* is upregulated in serum exosomes from patients with aggressive, invasive PCa, stating that high *miR-1246* is significantly associated with lymph node metastasis, distinguishing cancer exosomes from those of normal or BPH patients [70]. It was discovered that exosomal *miR-6880-5p* was significantly reduced in patients with PCa, with the most pronounced reduction observed in those with treatment-resistant, progressive disease. It can therefore be deduced that *miR-6880-5p* has a tumor-suppressive role. Functional studies have demonstrated that the enforced expression of *miR-6880-5p* in CRPC leads to reduced cellular proliferation, migration, and invasion. In line with these, there was suppression of oncogenic pathways, such as Ras, MAPK, and ERBB, as well as activation of tumor-suppressive pathways, including FOXO, ferroptosis, and autophagy. These pieces of evidence suggest that *miR-6880-5p* could potentially contribute to the regression of CRPC and can therefore be developed as a diagnostic and prognostic biomarker that can be tested using liquid biopsies [71]. Meanwhile, *miR-855-3p*, which has been identified in recent years within the peripheral blood of CRPC cases, has been shown to increase and play a pivotal role in tumor growth mechanisms, indicating a possible role in progression processes. Circulating *miR-222-3p* has emerged as a potential predictor of docetaxel resistance in metastatic CRPC cases, with higher plasma levels observed in patients who did not respond to docetaxel treatment and a reported ROC AUC of 0.76 (95% CI, 0.55–0.97). The above miRNAs consolidate the role and importance of liquid biopsies within monitoring aggressive and resistant phenotypes within advanced prostate malignancies [72]. Furthermore, multi-miRNA prognostic panels have been developed. A urinary tri-miRNA panel (*miR-125b-5p*, *let-7a-5p*, *miR-151-5p*) predicted time to biochemical recurrence after radical prostatectomy independently of standard clinicopathological parameters, illustrating the first urine-based miRNA signature predicting prognosis in PCa to gain validation. The concerned miRNAs have already demonstrated the ability to facilitate disease progression by inhibiting apoptosis, increasing proliferative rates, and enhancing migratory and invasive potential [73]. Another model, the PCa Metastasis Risk Scoring Model (PCa-MRS), which included *miR-21*, *miR-451*, and *miR-636*, along with preoperative PSA, demonstrated higher accuracy (AUC = 0.925) and predicted poorer biochemical recurrence-free survival. The model was derived from the analysis of urinary exosomal miRNAs associated with metastatic disease, in which the upregulation of *miR-21* and *miR-451*, and the downregulation of *miR-636*, remained significant in multivariate analysis as independent factors [74]. A four-miRNA panel in serum (*miR-17*, *miR-20a*, *miR-20b*, *miR-106a*) can distinguish high-risk and low-risk patients after prostatectomy. The expression of these miRNAs was associated with aggressive pathological characteristics and the time to biochemical recurrence. Functional studies confirmed that these miRNAs induce an aggressive disease phenotype when overexpressed, thereby validating their role in predicting PCa prognosis [60] (Figure 2).

Table 2 highlights key miRNAs associated with PCa prognosis, detailing their biological sources, regulatory pathways, correlations with disease aggressiveness or progression, and prognostic relevance across various clinical cohorts.

## 4. miRNAs for Treatment Monitoring and Therapeutic Response

### 4.1. MiRNA Dynamics with Systemic Therapy

Circulating miRNAs show dynamic fluctuations in response to systemic therapies, particularly ADT. Current evidence suggests that key miRNAs involved in ADT response are directly linked to mechanisms of resistance and progression to CRPC, including the regulation of testosterone signaling, the PI3K/AKT pathway, and the VEGF pathway. Numerous studies have identified miRNAs whose expression levels change during pre-, on-, and post-ADT phases, reflecting both therapeutic efficacy and progression to CRPC. The following evidence integrates clinical observations with a mechanistic perspective on how to distinguish the behavior of predictive biomarkers from the downstream effects of therapy. Among these, *miR-21*, *miR-125b*, *miR-23b*, *miR-27b*, and *miR-221* are frequently reported as key modulators associated with treatment outcomes and disease progression. A meta-analysis of available data demonstrates that these five miRNAs represent the most promising predictors of ADT response and are functionally implicated in critical processes, such as proliferation, apoptosis, EMT, angiogenesis, and androgen independence evolution [76]. These findings highlight the potential of circulating miRNA signatures as real-time, minimally invasive biomarkers for monitoring therapeutic response.

Distinct miRNA alterations characteristically accompany ADT and the emergence of castration resistance. For example, *miR-21* and *miR-125b* are consistently upregulated following ADT, and their increase has been linked to treatment resistance and the development of CRPC [76,77,78]. Similarly, upregulation of *miR-221* correlates with ADT resistance. These three miRNAs, together with *miR-23b/-27b*, these miRNAs comprise a leading predictive panel for ADT response identified through integrated meta-analyses of clinical and bioinformatic studies [76]. In contrast, *miR-23b/-27b* are often downregulated in CRPC, linking their reduced expression to disease progression despite therapy [76,79,80]. Consistent with this pattern, *miR-27a*—another component of the same *miR-23/27* cluster—has also been shown to be downregulated during CRPC development via PI3K signaling and aberrant AR activity, further supporting the tumor-suppressive role of this miRNA family in advanced disease [79]. Additionally, circulating miRNAs such as *miR-375* and *miR-141* increase progressively with advancement to metastatic CRPC, providing complementary evidence that the ADT-resistant state is accompanied by coordinated shifts in tumor-derived miRNA expression [77]. Other miRNAs, such as *miR-205* and *miR-92b*, are found at lower levels in CRPC and are associated with tumor progression [81]. Novel candidates, including *miR-3195*, *miR-3687*, and *miR-4417*, show marked upregulation in CRPC compared with primary Pca, demonstrating strong discriminatory power between CRPC and primary tumors and exhibiting functional involvement in cell migration and invasion, positioning them as potential biomarkers for late-stage progression [81,82]. Additionally, CAFs-derived exosomal *miR-146a-5p* has been observed to decrease following ADT, and this loss promotes metastasis through activation of the EGFR/ERK pathway by enhancing EMT, migration, and invasion of Pca cells under castration conditions [22]. Resistance training induces changes in miRNAs that target muscle and metabolic functions in patients with Pca undergoing ADT. Affected miRNAs include *miR-1*, *miR-29b*, and *miR-133a*, all of which showed significantly higher plasma levels in the exercise group compared with controls. These alterations occur alongside documented improvements in lean body mass, strength output, and metabolic parameters during resistance-training interventions combined with ADT. Myogenic miRNAs, such as *miR-1*, *miR-29b*, and *miR-133a*, are known to play a role in muscle development and regeneration. Together, these data indicate that resistance training–induced modulation of myogenic miRNAs reflects beneficial adaptations in muscle and metabolic function during ADT [83]. Additionally, certain miRNAs have been identified as predictors of treatment efficacy for AR pathway inhibitors, such as abiraterone. Specifically, *miR-152-3p*, *miR-411-5p*, and *miR-34a-3p* show high expression levels in responders compared with non-responders, thereby attaining area under curve values ranging from 0.67 to 0.70 [84]. The aforementioned miRNAs are known for participating in the signaling pathway related to transforming growth factor-beta and p53 resistance. Such findings have opened a new avenue for monitoring the efficacy of the treatment on a real-time basis [84,85].

These dynamic miRNA changes provide a comprehensive approach to monitoring treatment response. Importantly, alterations in miRNA expression occur early during ADT and AR pathway inhibitors, often preceding detectable clinical evidence of therapeutic resistance [75]. First, they enable the early detection of resistance: miRNAs, including *miR-375*, *miR-141*, *miR-378*, and *miR-409-3p*, have been shown to shift in expression prior to ADT failure, reflecting the emergence of castration-resistant or hormone-refractory phenotypes [75,76]. Second, because circulating miRNAs can be repeatedly measured in blood, they serve as non-invasive biomarkers suitable for longitudinal monitoring and may reflect ongoing molecular changes, such as the activation of PI3K/AKT, EGFR, and NF-κB, as well as other survival pathways that contribute to treatment resistance [76,77,83]. Taken together, their predictive value supports personalized therapeutic strategies, wherein treatment regimens can be modified in real-time based on patient miRNA profiles and network-level miRNA alterations, potentially improving patient outcomes and enabling early intervention before the full development of castration-resistant prostate cancer.

Beyond ADT, the expression of circulating miRNA also plays a pivotal role in response to other systemic interventions. Several studies indicate that multi-miRNA signatures measured at the time of radical prostatectomy can distinguish individuals with early versus late (BCR, supporting their potential use as prognostic tools at surgery [86,87,88]. For instance, 37 differentially expressed miRNAs, including *miR-1*, *miR-133B*, *miR-221*, and *miR-449A*, have been associated with recurrent PCa after surgery, and miRNA-based classifiers could predict BCR with high accuracy (up to 97% in Gene Expression Omnibus (GEO) datasets) [86]. Additionally, *miR-10b* has been validated as an independent marker of early relapse, demonstrating a role in promoting migration and metastasis, while *miR-96* expression correlates with GS and biochemical relapse, reinforcing its prognostic relevance [87,88]. This evidence supports the potential of miRNA signatures as prognostic tools at the point of surgical intervention; however, their longitudinal utility for post-prostatectomy monitoring of BCR still requires further validation in prospective studies [86,87,88] (Figure 2).

### 4.2. Markers of Treatment Resistance

Baseline circulating miRNA signatures have emerged as promising indicators of patients who are most likely to develop resistance to first-line ADT. Mechanistic analyses suggest that *miR-125b* and *miR-21* contribute to survival and aggressive behavior, whereas *miR-23b/-27b* act as tumor suppressors by inhibiting proliferation, migration, and promoting apoptosis through targets such as MAP2K4 [76,79]. Indeed, *miR-221* appears to be linked to androgen-independent growth. Notably, *miR-27a* is downregulated in CRPC via aberrant AR and PI3K/AKT signaling, leading to de-repression of *MAP2K4*, which functions as an oncogene in PCa [26]. As these miRNAs target AR and the survival pathways of PI3K/AKT signaling, and others mediated by the survival factor VEGF, multi-miRNA signatures might offer better predictive performance than single-miRNA signatures. This network of miRNA–gene interactions provides a mechanistic framework for understanding resistance and may inform future combined therapeutic strategies [76,79]. Collectively, these miRNAs provide a mechanistic framework for understanding resistance (Table 2), though validation in prospective trials is required.

Similarly, in the context of BCR, upregulation of the *miR-17–92* cluster is associated with shorter time to BCR, indicating its value as a prognostic biomarker for recurrence risk [89]. Whereas circulating tumor DNA (ctDNA) harbors genome-based drivers of resistance, such as AR pathway potentiation, clonal rearrangements, and epigenetics, circulating miRNA offers additional insight into the regulatory pathways of chemoresistance. Though the integration of these strategies has not been assessed directly among the cited references, their respective mechanistic roles point toward the potential of combined analysis of ctDNA and miRNA profiles for revealing a more complete landscape of resistance development [90,91,92,93,94].

Taken together, baseline and dynamic miRNA profiles represent a robust layer of information for predicting response and resistance to treatment in PCa. While individual miRNAs, such as *miR-125b* and *miR-21*, highlight key oncogenic pathways, composite signatures and multi-analyte approaches (a miRNA panel plus ctDNA) offer superior predictive power. These findings underscore the translational potential of miRNAs for stratifying patients, guiding early therapeutic adjustments, and informing decisions on next-line treatments in advanced PCa.

### 4.3. miRNAs as Predictive and Pharmacodynamic Biomarkers

Circulating miRNAs have recently been proposed as potential predictive and early response biomarkers of systemic treatment outcomes in CRPC. High pretreatment expression of the *miR-200* family of miRNAs, particularly *miR-200b*, is associated with poor docetaxel response and shorter OS, possibly through enhanced metastatic colonization via inhibition of SEC23A-mediated secretion of metastasis-suppressive proteins. Similarly, low or unchanged levels of the *miR-17* family of miRNAs, *miR-20a*, after treatment are predictive of poor PSA response and shorter OS, potentially reflecting increased macrophage differentiation and inflammation-mediated chemoresistance. Crucially, multivariate Cox regression analyses showed that pretreatment *miR-200b* and changes in *miR-20a* after treatment were both independent predictors of OS. Additionally, decreased *miR-222* levels after docetaxel treatment were associated with a poor outcome, possibly due to the upregulation of angiogenesis via its target KIT, despite its variable tumor-suppressive or oncogenic roles depending on the cellular context. These findings highlight that circulating miRNAs can serve as both baseline predictors of chemotherapy sensitivity and dynamic indicators of treatment response in CRPC, providing mechanistic insight into docetaxel resistance and potential therapeutic targets [54] (Figure 2).

Apart from chemotherapy, circulating miRNA signatures have potential utility for predicting the likelihood of BCR after prostatectomy. Meta-analyses of recurrent PCa datasets indicate that deregulated miRNA signatures, including overexpressed *miR-125A-5p* and *miR-199a-3p*, can predict the probability of recurrence by integrating multiple datasets and identifying common differentially expressed miRNAs across studies. Functional enrichment and network analyses of these DE miRNAs suggest their involvement in the regulation of epithelial cell proliferation, tissue morphogenesis, and canonical cancer pathways, with hub genes such as *CTNNB1*, *MYC*, *MAX*, and *SREBF1* mediating key oncogenic processes that may influence recurrence risk [86]. On the other hand, the CAF-related miRNA signatures of *miR-1258* and *miR-133b* have been incorporated into prognostic models using computational algorithms such as LASSO, SVMs, Random Forest, and penalized regression to predict BCR and assess tumor microenvironment characteristics. These CAF-related miRNAs influence tumor cell proliferation, migration, and colony formation, and their modulation, both in vitro and in vivo, impacts tumor growth, highlighting potential targets for therapeutic intervention [95]. Likewise, highly expressed levels of the *miR-17-92* cluster have been reported as predictors of shorter BCR-free survival times. The *miR-17-92* cluster functions as an oncogenic polycistronic miRNA unit that promotes proliferation, suppresses apoptosis, and is androgen-regulated in PCa cells, making it a reliable prognostic biomarker for post-prostatectomy biochemical recurrence [89]. Collectively, current evidence establishes miRNAs as robust predictive and pharmacodynamic biomarkers in prostate cancer, providing clinically actionable information both before and during therapy. Baseline miRNA profiles, such as *miR-200b* and members of the *miR-17* family, predict chemotherapy sensitivity and survival outcomes. In contrast, dynamic treatment-associated changes in miRNAs, including *miR-20a* and *miR-222*, reflect pharmacodynamic responses linked to resistance mechanisms, including inflammation, altered secretion pathways, and angiogenesis. Beyond therapy monitoring, integrated miRNA signatures also stratify the risk of recurrence following prostatectomy (Figure 2).

Table 3 summarizes key miRNAs investigated for treatment monitoring and response assessment across ADT, chemotherapy, and recurrence settings, highlighting their biological sources, regulatory targets, clinical contexts, and associated implications.

## 5. Limitations and Specificity Considerations of Circulating miRNAs in PCa

Notwithstanding, several biological and analytical complexities must be considered to avoid overestimating diagnostic specificity. In the context of early disease, only a small proportion of circulating EVs are of direct prostatic origin. In contrast, the measurement of circulating EV-associated miRNAs is highly dependent on the fluid used (serum, plasma, urine, or semen), the EV isolation technique, and the normalization methodology and detection platform (e.g., qPCR versus next-generation sequencing (NGS)), collectively limiting cross-study comparisons [96]. Furthermore, significant pre-analytical variability, including sample collection methods, storage conditions, centrifugation protocols, and potential confounding factors such as hemolysis, must be rigorously controlled across cohorts. The specificity of a limited set of miRNAs, most notably *miR-98-5p* and *miR-326*, is apparently increased, characterized by consistent plasma overexpression with high diagnostic accuracy (AUC ≈ 0.88), reduced in most other malignancies, although not a characteristic phenomenon of circulating miRNA species [13]. Most of the miRNAs cited within the current review, such as *miR-141*, *miR-375*, *miR-21*, members within the *miR-200* family, and *miR-125b*, are consistently reported as aberrant within the context of PCa but are also similarly aberrated within other malignancies, including breast, colorectal, lung, and hepatocellular cancers, apart from non-malignant conditions such as inflammation, neurologic disorders, and systemic conditions, a phenomenon consistent with a regulatory paradigm that is pleiotropic and context-dependent [11,12]. The lack of absolute specificity is, furthermore, complicated by observations demonstrating that several miRNAs are reciprocally differentially expressed within tumor versus circulating fractions, most notably exemplified by *miR-326* and *miR-375*, consistent with the existence of selective secretion/export mechanisms [20]. In addition to tumor-derived signals, treatment-related and systemic factors substantially influence circulating miRNA profiles, which describe the scope of this review. Induced by androgen-deprivation therapy are the increased oncogenic expression of *miR-21*, *miR-125b*, and *miR-221*, but reduced tumor suppressive expression of *miR-23b/-27b*, reflecting tumor evolutionary adaptation consistent with the development of particular types of castration-resistant PCa [76,83]. Likewise, physiological responses, such as resistance exercise, affect the abundance of myogenic miRNAs (*miR-1*, *miR-29b*, *miR-133a*) in the circulation, irrespective of tumor burden [83]. In contrast, clinical comorbidities, such as bone metastases, obesity, and metabolism, are linked to changes in the circulating abundance of *miR-218-5p* [64], *miR-214* [41], *miR-107* [66,67], and *miR-181a-5p* [65], which themselves add to the complexities in disease-specific interpretation. The impact of such factors thus emphasizes that circulating miRNAs are capable of integrating multiple signals from tumor biology, therapeutics, host physiology, and the tumor microenvironment.

It is therefore evident that interpretation of a single miRNA marker is highly susceptible to false positives and negatives, making single-miRNA tests unsuitable for providing adequate specificity for PCa, particularly in the diverse real-world environment [13]. On the contrary, a uniform thread throughout the literature cited in this review clearly indicates that the highest utility of miRNAs exists within multi-miRNA signatures, when supplemented with proven ancillary factors such as preoperative evaluation of PSA, GS, imaging, and disease stage. For robust clinical translation, future studies must standardize the use of appropriate internal controls (e.g., spike-in sequences or consensus endogenous references) to account for variability introduced by RNA extraction efficiency and reverse transcription steps. That is, multi-miRNA signatures, such as plasma signatures for the detection of PCa within four miRNAs, five miRNA signatures that are apt for identifying extremely high-risk disease, and models predicting treatment failure capable of assessment within the urine or exosomes [40,50,54], clearly underscore a distinct advantage for identifying a high degree of certainty concerning disease treatment, growth, failure, as well as relapse when contrasted with a single miRNA marker concerning assistive assessments within PCa treatment, growth, failure, and relapse [26,27,28]. Hence, translational studies aimed at precise assessment of circulating miRNAs require evaluation in large, well-characterized clinical cohorts that include patients with relevant disease states and comorbidities, along with disease- and comorbidity-matched controls and rigorous standardization of both preanalytical and analytical procedures [96]. In this respect, the use of circulating miRNAs should be viewed as a complementary tool that enhances the detection, prognostic potential, and real-time monitoring of treatment for PCa, but not as a disease-specific marker per se.

## 6. Conclusions and Future Directions

Liquid Biopsy miRNAs have emerged as pivotal components of PCa research as multifaceted biomarkers for early detection, prognosis, and treatment monitoring. In this review, we edited and synthesized current knowledge on how miRNAs can complement or surpass traditional tools, such as PSA. Circulating miRNAs, such as miR-141 and *miR-375*, show strong potential in identifying aggressive or metastatic disease at diagnosis, whereas others, like *miR-21* and the *miR-17-92* cluster, signal poor outcomes and therapeutic resistance. Unlike static tissue markers, miRNAs can be repeatedly sampled from blood, urine, or semen, providing a dynamic view of tumor evolution. This real-time molecular insight enables earlier intervention; for example, rising levels of oncomiRs during ADT can a warning of treatment failure well before PSA or imaging changes become apparent. By assembling panels of multiple miRNAs, clinicians could achieve more accurate risk stratification and monitoring than any single biomarker alone, reflecting the heterogeneity of PCa. The translation path for miRNA-based liquid biopsies is hindered by a high degree of methodological variability, as mentioned in Section 5 of this review, which must be addressed before their routine clinical implementation can be achieved. In addition, standardization remains a primary limitation, as variability in sample type (plasma versus serum), isolation strategies, and normalization methods continues to hinder cross-study comparison and reproducibility. Furthermore, inconsistent assay calibration complicates the evaluation of clinical utility and evidence for individual miRNA candidates. In addition, analytical complexity arises from the fact that only a small fraction of circulating small extracellular vesicles (SEVs) originate from prostate tumor cells, particularly in patients with localized or early-stage disease, making detection of tumor-specific vesicular miRNA signals inherently challenging. Emerging approaches, such as PSMA-targeted enrichment of prostate-derived SEVs using aptamer-functionalized superparamagnetic beads, have demonstrated improved sensitivity and specificity over total SEV analysis or antibody-based capture methods. However, miRNA loading into SEVs is an active, regulated process, meaning that intracellular miRNA expression does not always correlate with vesicular content [96]. An additional interpretive challenge arises from the observation that certain miRNAs exhibit oncogenic associations in circulation while demonstrating tumor-suppressive functions within prostate tissue. This apparent discordance underscores the complexity of miRNA biology and highlights the unique interpretive considerations inherent to liquid biopsy-based biomarkers. Circulating miRNAs may originate from multiple sources, including direct release from tumor cells, secretion via EVs, treatment-induced cellular stress, immune cell activation, or broader systemic physiological responses. Consequently, elevated circulating levels of a given miRNA do not necessarily reflect its intracellular function within tumor cells. Studies relying exclusively on blood-based miRNA profiling may therefore capture a composite signal influenced by tumor burden, therapeutic intervention, and host response. In contrast, tissue-based analyses provide direct insight into tumor-intrinsic regulatory networks. Integrative studies combining paired tissue and liquid biopsy analyses will be essential to disentangle these contributions and refine the clinical interpretation of circulating miRNAs in prostate cancer. These factors underscore the importance of selecting highly prostate-specific miRNA markers and refining SEV isolation and quantification technologies to enhance diagnostic and predictive accuracy. In parallel, most miRNA biomarkers identified to date require validation in large, prospective clinical trials. Retrospective studies and small cohorts have identified numerous candidate miRNAs; the next step is to evaluate these markers in multi-center settings with diverse patient populations, ensuring that they reliably improve decision-making (e.g., avoiding unnecessary biopsies or tailoring therapy) compared to existing standards. Regulatory approval of miRNA diagnostic kits will hinge on such validation, as well as demonstrating cost-effectiveness and clinical utility. miRNAs have emerged as promising biomarkers due to advances in large-scale profiling, analytical platforms, and standardized workflows, which have improved the reliability of circulating miRNA signatures for prostate cancer diagnosis, risk stratification, and disease monitoring. The coming years are likely to witness a deeper integration of miRNA analytics with other emerging technologies in PCa management. For early detection, we anticipate that miRNA signatures could be combined with advanced imaging or other blood-based markers (such as circulating tumor cells, cell-free DNA, or proteomic profiles) to create robust multi-modal screening tools. The synergy between miRNAs and traditional biomarkers, such as PSA, might significantly enhance accuracy. For instance, a high PSA accompanied by a high-risk miRNA panel could prompt immediate biopsy, whereas an elevated PSA with a normal miRNA profile might warrant watchful waiting. These emerging miRNA signatures can be evaluated in the context of existing commercially available urinary PCa tests, including PCA3, SelectMDx, and ExoDx Prostate IntelliScore. Although these assays have achieved clinical adoption, miRNA panels offer a complementary advantage by leveraging blood-based sampling, which may capture distinct aspects of tumor biology. While multicohort studies have demonstrated promising performance for circulating miRNAs such as *miR-141* and *miR-375*, FDA or CE approval for individual miRNAs or miRNA-based liquid biopsy platforms in PCa remains lacking. Establishing regulatory approval, therefore, represents an important next step for the field, particularly given the successful clinical integration of existing commercial assays. In the prognostic realm, miRNA profiles might help delineate which localized cancers harbor lethal potential, guiding decisions between active surveillance and definitive treatment. There is also growing interest in whether changes in circulating miRNAs after surgery or radiation can serve as ultra-early indicators of minimal residual disease or imminent recurrence, potentially years before a rise in PSA. Next-generation sequencing and digital droplet PCR are making it feasible to quantify dozens of miRNAs simultaneously with high sensitivity and reproducibility. Machine learning algorithms are increasingly applied to identify patterns within complex miRNA datasets that humans might miss—for example, algorithms could discover composite miRNA signatures that optimally predict treatment response or long-term survival. As data accumulates, we may see the development of artificial intelligence (AI)-driven decision support systems that incorporate miRNA levels alongside clinical factors to recommend personalized treatment plans. Another promising avenue is therapeutic targeting of miRNAs. The same miRNAs that indicate poor prognosis or resistance (such as *miR-21* or *miR-125b*) could be targeted with anti-miR oligonucleotides to restore treatment sensitivity, a strategy already being tested in preclinical models. Conversely, tumor-suppressive miRNAs, such as *miR-34a* or *miR-143*, could be delivered to tumors using nanoparticle vehicles, as early studies have shown, opening a new class of miRNA-based therapeutics. In the future, patient-derived liquid biopsies could support both cancer diagnosis and the rational selection of miRNA-based therapeutic strategies targeting tumor-specific molecular features. In conclusion, miRNAs in liquid biopsies represent a transformative frontier in prostate cancer care. Their unique stability and capacity to reflect tumor biology enable applications ranging from non-invasive screening and more accurate prognostication to real-time monitoring of treatment efficacy and resistance. Embracing miRNA-based tools will propel prostate oncology toward a more precise and personalized paradigm. Realizing this potential will require concerted efforts to surmount technical hurdles and validate clinical benefit. As large-scale collaborations and technological innovations continue to unfold, there is an optimistic expectation that circulating miRNAs will transition from the bench to the bedside, improving outcomes for patients with PCa through earlier detection, better risk stratification, and adaptive biomarker-guided therapy decisions. Ultimately, integrating miRNA-based assays with complementary biomarkers within defined clinical contexts may enable more precise patient stratification, reduce overtreatment, and inform timely therapeutic decision-making in prostate cancer.

## Figures and Tables

**Table 1:** miRNAs in PCa detection.

miRNA	Liquid Biopsy	Target	Sources	Diagnostic Role	Refs.
*miR-9-3p*	Serum (blood)	Not specified	Cell-free circulating miRNA	Increases in PCa	[14]
*miR-19a-3p*	Blood	*SOX4*; EMT regulators (*MMP2*, *MMP9*, *N-cadherin*, *Vimentin*, *α-SMA*, *E-cadherin*)	Cell-free circulating miRNA	Tumor-suppressive function; reduces invasion and EMT	[16]
*miR-21*	Urinary pellets, urinary exosomes	*PTEN*, *PDCD4*, *SPRY1*; AR signaling; EMT pathways	Pellets and exosomes	Increases in non-metastatic and metastatic PCa	[18,29,32]
*miR-34a-5p*	Urine	Not specified	Urinary exosomes	Decreases in PCa	[32]
*miR-92a-1-5p*	Urine	Not specified	Urinary exosomes	Decreases in PCa	[32]
*miR-98-5p*	Plasma (blood)	Not specified	Cell-free circulating miRNA	Increases in PCa; relatively PCa-specific; possible prognostic relevance	[13]
*miR-126-3p*	Urine	Not specified	Urinary EVs	Increases; significant early detection biomarker; better performance than PSA	[31]
*miR-1290*	Urine	Not specified	Urinary EVs	Increases, higher in metastatic PCa	[25]
*miR-141*	Urine pellets/EVs	Not specified	Urinary pellets and EVs	Increases; correlates with the Gleason score	[11,12,18]
*miR-142-3p/5p*	Semen	APC → Wnt/β-catenin pathway	Semen exosomes	Increases; improves PSA specificity in PCa vs. BPH	[38]
*miR-143-3p*	Urine	Not specified	Urinary exosomes	Decreases in PCa	[38]
*miR-145*	Urine	*FSCN1*; EMT regulators	Urinary EVs/exosomes	Increases (especially GS ≥ 8); improves PSA diagnostic performance	[26,40]
*miR-146a-5p*	CAF-EVs	ROCK/Caspase-3; EGFR/ERK pathways	Tissue and CAF-derived exosomes	Decreases in high GS and metastatic PCa	[21,22]
*miR-152-3p*	Plasma (blood)	Not specified	Cell-free circulating miRNA	Increases as part of the diagnostic 4-miRNA plasma panel	[13]
*miR-183*	Blood/tissue	PSA 3′UTR (direct regulation of PSA expression)	Prostate tissue and circulation	Increases; correlates with PSA, higher grade, and progression	[15]
*miR-196a-5p*	Urine	ERG, HOX genes	Urinary exosomes	Decreases; high sensitivity for distinguishing PCa from normal tissue.	[32]
*miR-200c*	Urine	EMT suppression pathways	Cell-free urine	Decreases in PCa	[33,34]
*miR-214*	Urine	Not specified	Urinary exosomes	Decreases in PCa	[18,41]
*miR-223-3p*	Semen	*TP53*, *FOXO1*, *CDK2*, *IGF1R*, *MDM2*, *CHUK*, *HSP90B1*	Semen exosomes	Increases; discriminates PCa from controls	[38]
*miR-326*	Plasma (blood)	HOTAIR-regulated tumor-suppressive pathways	Cell-free circulating miRNA	Increases in plasma; tissue downregulation; linked to disease progression	[13]
*miR-330-3p*	Serum (blood)	Not specified	Cell-free circulating miRNA	Increases in PCa	[14]
*miR-342-3p*	Semen	*IGF1R*, *E2F1*, *ATF4*, *IKBKG*	Semen exosomes	Increases; predicts GS ≥ 7 with PSA	[38]
*miR-345-5p*	Serum (blood)	Not specified	Cell-free circulating miRNA	Increases; diagnostic and treatment response candidate	[14]
*miR-374b-5p*	Semen	Not specified	Semen exosomes	Increased; predicts GS ≥ 7	[38]
*miR-375*	Blood, urine	*CCND2*; ZEB1/miR-375/YAP1 signaling axis	Tissue, circulating miRNA, urinary EVs	Increases; associated with GS, stage, and metastasis; strong diagnostic performance	[20,28,42]
*miR-4289*	Plasma (blood)	Not specified	Cell-free circulating miRNA	Increases as part of the diagnostic plasma panel	[13]
*miR-483-5p*	Urine	Angiogenesis and metastasis pathways	Cell-free urine fraction	Increased; enhances diagnostic performance with PSA	[30]
*miR-501-3p*	Urine	ERG, HOX genes	Urinary exosomes	Decreases in PCa	[32]
*miR-574-3p*	Urine	Not specified	Urinary EVs	Increases; improves multi-miRNA diagnostic panel	[27]

**Table 2:** miRNAs in PCa prognosis.

miRNA	Liquid Biopsy	Target	Sources	Diagnostic Role	Refs.
*let-7a-5p*	Urine	Not specified	Urinary exosomal	Part of the tri-miRNA panel predicting biochemical recurrence	[73]
*let-7i*	Plasma/serum	Not specified	Cell-free	Decreases with increasing GS; discriminates low vs. high-risk disease	[49,75]
*miR-17*	Serum	Not specified	Cell-free	Part of a four-miRNA panel associated with aggressive pathology and early recurrence	[60]
*miR-18b-5p*	Serum/plasma	Not specified	Cell-free	Decreases with increasing GS	[50]
*miR-20a*	Serum/plasma	*E2F1*	Cell-free	Elevates in GS 7–10 and high CAPRA scores; identifies aggressive disease	[40]
*miR-20b*	Serum/plasma	Not specified	Cell-free	Component of recurrence and high-risk panels	[60]
*miR-21*	Plasma, serum, PBMCs, urine exosomes	*PTEN*, *TPM1*, *PDCD4*	Cell-free, PBMC, exosomal	Elevates in aggressive PCa, CRPC, recurrence, metastasis, and poor OS	[40,54,58,63,74]
*miR-25-3p*	Serum/plasma	Not specified	Cell-free	Reduces with increasing GS	[50]
*miR-26a*	Serum/plasma	*EZH2*	Cell-free	Higher in low-risk disease; inverse correlation with malignancy	[50]
*miR-26b-5p*	Serum/plasma	*EZH2*	Cell-free	Decreases with increasing GS; distinguishes pGS6/7 vs. pGS8	[50,55]
*miR-34a*	Urine	Not specified	Cell-free	Reduces in metastatic disease; weak association with OS	[62]
*miR-106a-5p*	Serum/plasma	PI3K/AKT pathway	Cell-free	Reduces in high-risk Pca; included in recurrence panels	[50,55]
*miR-107*	Plasma	Not specified	Cell-free	Upregulates in CRPC and stage IV; diagnostic adjunct to PSA	[66,67]
*miR-1246*	Serum exosomes	Not specified	Exosomal	Elevates in LN-positive and aggressive disease	[70]
*miR-125b-5p*	Urine	Not specified	Urinary exosomal	Part of the tri-miRNA urinary recurrence panel	[73]
*miR-128*	Serum & tissue	*BMI1*, *E2F3*, *DCX*, *NTRK3*	Cell-free & tissue-derived	Decreases levels predict metastasis and reduced recurrence-free survival	[61,63]
*miR-1290*	Plasma exosomes	Not specified	Exosomal	Elevates in CRPC; predicts poor OS	[42]
*miR-135a*	Serum	PI3K/AKT pathway	Cell-free	Component of validated VHR aggressive disease signature	[50,55]
*miR-139-5p*	Peripheral blood	Not specified	Cell-free	Associates with GS > 7, high PSA, and advanced pathological stage	[57]
*miR-141*	Plasma/serum	Not specified	Cell-free	Elevates in high-grade and lymph node-positive disease	[42,48]
*miR-145*	Plasma/serum	*KIF23*, *CCNA2*	Cell-free	Increases in intermediate/high-risk D’Amico groups	[40,52]
*miR-151-5p*	Urine	Not specified	Urinary exosomal	Tri-miRNA panel component for biochemical recurrence	[73]
*miR-181a-5p*	Serum exosomes	Not specified	Exosomal	Elevates in bone-metastatic Pca; diagnostic marker	[65]
*miR-200 family (miR-200c)*	Plasma/serum	*SEC23A*	Cell-free	High levels predict metastatic colonization and poor OS in CRPC	[53,54]
*miR-214*	Serum	*PTEN*	Cell-free	Increases in bone-metastatic and poorly differentiated Pca	[41]
*miR-218-5p*	Serum	Not specified	Cell-free	Reduces in bone metastasis; predicts shorter metastasis-free survival	[64]
*miR-221*	Plasma/serum	*p27Kip1*, *ARHI*, *DVL2*	Cell-free	OncomiR linked to invasion and aggressive disease	[40,51]
*miR-331-3p*	Blood	*CDCA5*	Cell-free	Cell-cycle regulator; downregulation promotes aggressive behavior	[52]
*miR-363-3p*	Serum/plasma	Not specified	Cell-free	Reduces with rising pGS; high-risk prognostic biomarker	[50,55]
*miR-375*	Plasma, serum, urine	*CBX7*, *YAP1*	Cell-free & exosomal	Elevates in aggressive, metastatic, and CRPC disease; predicts poor OS	[18,42,48,53]
*miR-433*	Serum	PI3K/AKT pathway	Cell-free	Part of VHR molecular signature	[50,55]
*miR-451*	Urine exosomes	Not specified	Exosomal	Component of Pca-MRS metastasis/recurrence risk model	[74]
*miR-605*	Serum	PI3K/AKT pathway	Cell-free	VHR disease signature biomarker	[50,55]
*miR-636*	Urine exosomes	Not specified	Exosomal	Downregulates in metastatic-risk Pca-MRS model	[74]
*miR-6880-5p*	Plasma exosomes	Ras/MAPK/ErbB regulators	Exosomal	Suppressed in CRPC; tumor-suppressive prognostic biomarker	[71]
*miR-855-3p*	Peripheral blood	Not specified	Cell-free	Elevates in CRPC and progressive disease	[72]
*miR-940*	Serum	*MIEN1*	Cell-free	Elevates in GS ≥ 7; improves PSA diagnostic accuracy	[59]

**Table 3:** miRNAs in treatment monitoring and therapeutic response.

miRNA Name	Source of Biopsy	Target of miR	Treatment Context	Change and Clinical Indication	Refs.
*miR-1*	Blood/plasma	Myogenic differentiation, Myogenic networks	ADT + resistance training, post prostatectomy	Upregulated, reflects improved lean mass and muscle regeneration during ADT, component of miRNA classifiers predicting BCR	[83,86]
*miR-10b*	Blood/plasma	Migration and metastasis pathways	Post-prostatectomy	Upregulated, independent early relapse marker	[87,88]
*miR-17–92 cluster*	Blood/plasma	MYC-regulated proliferation and apoptosis control	Post prostatectomy (BCR risk)	Upregulated, predicts shorter BCR-free survival	[89]
*miR-20a*	Blood/plasma	Immune differentiation and inflammatory chemoresistance	Chemotherapy (docetaxel)	Low or unchanged post-therapy, predicts poor PSA response and shorter OS	[54,60]
*miR-21*	Blood/plasma	AR signaling, PI3K/AKT, VEGF pathways	ADT → CRPC	Upregulated, predicts ADT resistance and CRPC development	[76,79]
*miR-23b*	Blood/plasma	*MAP2K4*, EMT suppression	CRPC	Downregulated, loss associated with progression and therapy resistance	[76,79]
*miR-27a*	Blood/plasma	*MAP2K4*; AR/PI3K signaling	CRPC	Downregulated, de-repression of oncogenic signaling in advanced disease	[26,79]
*miR-27b*	Blood/plasma	*MAP2K4*, EMT suppression	CRPC	Downregulated, tumor-suppressive loss associated with CRPC	[76]
*miR-29b*	Blood/plasma	Myogenesis and metabolic regulation	ADT + resistance training	Upregulated, biomarker of beneficial metabolic adaptation	[83]
*miR-92b*	Blood/serum	Proliferation and invasion pathways	CRPC	Downregulated, indicator of aggressive transition to CRPC	[81]
*miR-96*	Blood/plasma	Androgen and proliferative signaling	Post prostatectomy	Upregulated, correlates with higher Gleason score and BCR risk	[88]
*miR-125A-5p*	Blood/plasma	Proliferation and recurrence pathways	Post prostatectomy	Upregulated, predicts the probability of biochemical recurrence	[86]
*miR-125b*	Blood/plasma	AR signaling and anti-apoptotic pathways	ADT → CRPC	Upregulated, marker of resistance and castration resistance	[76]
*miR-133a*	Blood/plasma	Muscle regeneration signaling	ADT + resistance training	Upregulated, reflects functional muscle recovery	[83]
*miR-133b*	Blood/plasma	CAF-mediated tumor growth signaling	Post prostatectomy	Altered expression, incorporated into prognostic BCR models	[86]
*miR-141*	Blood/serum	EMT and metastatic networks	ADT → mCRPC	Upregulated, marker of advanced disease and resistance	[75,76,77]
*miR-146a-5p*	Blood/plasma exosomes	EGFR/ERK and EMT regulation	ADT	Downregulated, loss promotes EMT-driven metastasis	[21,22]
*miR-199a-3p*	Blood/plasma	Morphogenesis and EMT regulation	Post prostatectomy	Upregulated, predictor of biochemical recurrence	[86]
*miR-200b*	Blood/plasma	SEC23A secretory pathway	Chemotherapy (docetaxel)	High baseline expression, predicts poor treatment response and shorter OS	[54]
*miR-205*	Blood/serum	ZEB1-mediated EMT regulation	CRPC	Downregulated, associated with tumor progression	[81]
*miR-221*	Blood/plasma	Androgen-independent growth signaling	ADT	Upregulated, correlates with castration resistance	[26,76,86]
*miR-222*	Blood/plasma	KIT signaling and angiogenesis	Chemotherapy (docetaxel)	Decreased post therapy, indicates an unfavorable treatment response	[54]
*miR-375*	Blood/serum	Neuroendocrine and survival pathways	ADT → mCRPC	Upregulated, early indicator of metastatic CRPC	[77]
*miR-378*	Blood/plasma	Metabolic and survival signaling	ADT	Altered early, early detection of emerging resistance	[75,76]
*miR-409-3p*	Blood/plasma	EMT and metastatic regulators	ADT	Altered early, predictive of hormone-refractory transition	[75,76]
*miR-449A*	Blood/plasma	Cell cycle control pathways	Post prostatectomy	Differential expression, predictor of biochemical recurrence	[86]
*miR-3195*	Blood/plasma	Migration and invasion signaling	CRPC	Upregulated, discriminates CRPC from localized PCa	[81]
*miR-3687*	Blood/plasma	Migration and invasion signaling	CRPC	Upregulated, biomarker of late-stage disease progression	[81]
*miR-4417*	Blood/plasma	Migration and invasion networks	CRPC	Upregulated, candidate biomarker of late-stage metastatic progression	[81]

## Data Availability

No new data were created or analyzed in this study.

## Abbreviations

ADT	Androgen-Deprivation Therapy
AR	Androgen Receptor
AUC	Area Under the Curve
BCR	Biochemical Recurrence
BPH	Benign Prostatic Hyperplasia
CAF	Cancer-Associated Fibroblast
CAPRA	Cancer of the Prostate Risk Assessment
CRPC	Castration-Resistant Prostate Cancer
CTC	Circulating Tumor Cell
ctDNA	Circulating Tumor DNA
DDR	DNA Damage Repair
DRE	Digital Rectal Examination
EMT	Epithelial–Mesenchymal Transition
EV	Extracellular Vesicle
GS	Gleason Score
HRPC	Hormone-Refractory Prostate Cancer
MET	Mesenchymal–Epithelial Transition
miRNA	microRNA
MRI	Magnetic Resonance Imaging
mCRPC	Metastatic Castration-Resistant Prostate Cancer
OS	Overall Survival
PCa	Prostate Cancer
PBMC	Peripheral Blood Mononuclear Cell
PET	Positron Emission Tomography
PI3K	Phosphoinositide 3-Kinase
PSA	Prostate-Specific Antigen
PSMA	Prostate-Specific Membrane Antigen
ROC	Receiver Operating Characteristic
SEV	Small Extracellular Vesicle
TRUS	Transrectal Ultrasound
UTR	Untranslated Region
VEGF	Vascular Endothelial Growth Factor
VHR	Very High-Risk
